# Electrophysiological Brain Changes Associated With Cognitive Improvement in a Pediatric Attention Deficit Hyperactivity Disorder Digital Artificial Intelligence-Driven Intervention: Randomized Controlled Trial

**DOI:** 10.2196/25466

**Published:** 2021-11-26

**Authors:** Rafael Medina, Jaime Bouhaben, Ignacio de Ramón, Pablo Cuesta, Luis Antón-Toro, Javier Pacios, Javier Quintero, Josep Antoni Ramos-Quiroga, Fernando Maestú

**Affiliations:** 1 Sincrolab Ltd Madrid Spain; 2 Laboratory of Computational and Cognitive Neuroscience Centre for Biomedical Technology Polytechnic University of Madrid Pozuelo de Alarcón Spain; 3 Faculty of Health Camilo Jose Cela University Villafranca del Castillo Spain; 4 Radiology Rehabilitation and Physiotherapy Complutense University of Madrid Madrid Spain; 5 Department of Experimental Psychology Faculty of Psychology Complutense University of Madrid Madrid Spain; 6 Department of Psychiatry University Hospital Infanta Leonor Madrid Spain; 7 Department of Psychiatry Hospital Universitari Vall d’Hebron Barcelona Spain; 8 Group of Psychiatry, Mental Health and Addictions Vall d’Hebron Research Institute Barcelona Spain; 9 Biomedical Network Research Centre on Mental Health Barcelona Spain

**Keywords:** ADHD, cognitive stimulation, magnetoencephalography, artificial intelligence, Conners continuous performance test, KAD_SCL_01, AI, cognitive impairment, attention deficit hyperactivity disorder, pediatrics, children, rehabilitation

## Abstract

**Background:**

Cognitive stimulation therapy appears to show promising results in the rehabilitation of impaired cognitive processes in attention deficit hyperactivity disorder.

**Objective:**

Encouraged by this evidence and the ever-increasing use of technology and artificial intelligence for therapeutic purposes, we examined whether cognitive stimulation therapy implemented on a mobile device and controlled by an artificial intelligence engine can be effective in the neurocognitive rehabilitation of these patients.

**Methods:**

In this randomized study, 29 child participants (25 males) underwent training with a smart, digital, cognitive stimulation program (KAD_SCL_01) or with 3 commercial video games for 12 weeks, 3 days a week, 15 minutes a day. Participants completed a neuropsychological assessment and a preintervention and postintervention magnetoencephalography study in a resting state with their eyes closed. In addition, information on clinical symptoms was collected from the child´s legal guardians.

**Results:**

In line with our main hypothesis, we found evidence that smart, digital, cognitive treatment results in improvements in inhibitory control performance. Improvements were also found in visuospatial working memory performance and in the cognitive flexibility, working memory, and behavior and general executive functioning behavioral clinical indexes in this group of participants. Finally, the improvements found in inhibitory control were related to increases in alpha-band power in all participants in the posterior regions, including 2 default mode network regions of the interest: the bilateral precuneus and the bilateral posterior cingulate cortex. However, only the participants who underwent cognitive stimulation intervention (KAD_SCL_01) showed a significant increase in this relationship.

**Conclusions:**

The results seem to indicate that smart, digital treatment can be effective in the inhibitory control and visuospatial working memory rehabilitation in patients with attention deficit hyperactivity disorder. Furthermore, the relation of the inhibitory control with alpha-band power changes could mean that these changes are a product of plasticity mechanisms or changes in the neuromodulatory dynamics.

**Trial Registration:**

ISRCTN Registry ISRCTN71041318; https://www.isrctn.com/ISRCTN71041318

## Introduction

Inhibitory control deficit is one of the core impairments in attention deficit hyperactivity disorder (ADHD) [1,2]. This deficit is directly related to the levels of impulsiveness present in the symptoms of ADHD [1,3-5] and produces difficulties in the everyday activities of those afflicted [6] while adversely affecting academic performance [7]. According to the literature reviewed, other impairments can be found in ADHD including the performance of cognitive processes, such as working memory [8], sustained attention [9,10], alternating attention [11], and planning [12,13].

In ADHD, the indices of inhibition, task switching, and emotional control appear to be related to relative power values of the alpha frequency band (7-13 Hz) in midline brain regions measured at resting state [14,15] and with performance in attentional tasks [16]. These patients consistently present a decrease in the alpha band in the central and posterior regions [17-24], as well as an increase in the theta frequency band (3-7 Hz) and the theta: beta ratio [17-21,25-28]. The decrease of the alpha band in regions that engage the default mode network (DMN; active network at resting state which includes the caudate nucleus, medial prefrontal cortex, posterior cingulate cortex, hippocampus, inferior parietal lobe, cerebellum, and precuneus) could modulate impairments in the functional connectivity of this network [29-33]. These impairments in the DMN also seem to be related to inhibitory control deficits [34,35].

Cognitive stimulation therapy appears to be effective in patients with ADHD [36]. The progressive increase of the workload in cognitive stimulation tasks is one of the main treatment dynamics of this type of therapy [37], and there are many examples of its effectiveness in ADHD and other disorders [38-42]. Its effectiveness seems to stem from the fact that these increases in the workload in cognitive tasks trigger an increase in long-distance connections supported by alpha and beta bands, and a decrease in short-distance connections supported by delta and theta bands [43-46].

Although the increase of the workload in cognitive stimulation tasks has shown promise in neurocognitive rehabilitation in children with ADHD, a case-based reasoning (CBR) system [47] that allows the adaptive workload to increase for each patient has never been used. The CRB system has been successful in various clinical areas [48-50], but its efficacy in a digital treatment for rehabilitation of neurocognitive alterations and its relation to electrophysiological dynamics and its efficacy in the rehabilitation of clinical alterations in ADHD remain unknown. With the aim of providing evidence, we examined whether a CRB digital training regimen would be effective in an ADHD child population after 12 weeks using the continuous performance test (CPT) inhibitory control measure as the main outcome. We hypothesized that after the intervention, the inhibitory control, as a core symptom of ADHD, would show a better performance and that this would be related to changes in the alpha band in the posterior regions and the DMN according to magnetoencephalography (MEG). We also tested whether treatment-produced changes in secondary outcomes would be related to ADHD and, finally, whether it could decrease the clinical symptoms associated with ADHD and change those behaviors related to executive functioning.

## Methods

The study was approved by the local ethics committee of the San Carlos Hospital (Madrid, Spain). All legal representatives of the participants gave their written informed consent to participate in the study. This clinical trial is registered in the ISRCTN registry (ISRCTN71041318).

### Participants

A total of 41 children diagnosed with combined-type ADHD (ADHD-C) were recruited (34 males). Contact with participants’ legal guardians was made through health facilities, schools, and associations in the community of Madrid. Research staff first contacted those private and public clinical centers asking for permission and agreement to recruit. The order in which centers were contacted was at random. The following recruitment actions were performed: emailing study information, phone calls, and teleconferences and webinars with legal guardians summarizing study information. Participants’ legal guardians who agreed to participate authorized communications with research staff. Eligibility criteria were checked by phone and email with legal guardians prior to visit 1. Before any other study activity, legal guardians read and signed an informed consent. There were no artificial intelligence (AI) requirements for the eligibility.

To be eligible, participants had to meet the following 5 criteria: (1) aged 8 to 11 years; (2) diagnosis of ADHD-C by an authorized professional (chartered psychiatrists at the medical college); (3) cessation of ADHD medication 3 days before each visit day, as, according to the technical specification of the drug methylphenidate (Concerta), it has a half-life of 3.5 hours (90% is excreted in urine and 1 to 35 in feces as a metabolite at 48-96 hours); (4) maintenance of the same level of medication during the at-home intervention period; and (5) compliance with the intervention protocol.

ADHD diagnosis was performed by accredited expert professionals following the *Diagnostic and Statistical Manual of Mental Disorders, Fifth Edition* (DSM-5) criteria. These diagnostic criteria were the same across all participants. The average time from ADHD diagnosis confirmation to study enrollment was 2.58 (SD 1.21) years.

Participants meeting any of the following 5 exclusion criteria were dropped from the trial: (1) the initiation or abandonment of behavioral therapies or psychoactive drugs during the at-home intervention period; (2) motor difficulties which made the use of the mobile device (tablet or smartphone) impossible; (3) use of psychoactive drugs (such as benzodiazepines) which could have acted as a confounding factor, presence or suspicion of substance abuse for the past 6 months; (4) presence of blindness or uncorrected visual acuity difficulties; and (5) any additional psychological diagnosis.

The inclusion criterion at the level of input data for the AI was a diagnosis of ADHD-C by an authorized professional in order to register the patient on the platform.

The use of other psychoactive drugs different from those approved by the Spanish Agency of Medicines and Medical Devices or European Medicines Agency for pediatric ADHD intervention (dextroamphetamine, levoamphetamine, lisdexamphetamine, methylphenidate, atomoxetine) also made participants ineligible for the study. The compliance was checked at the beginning and the end of the participant’s participation through the child's legal guardians.

**Figure 1:**
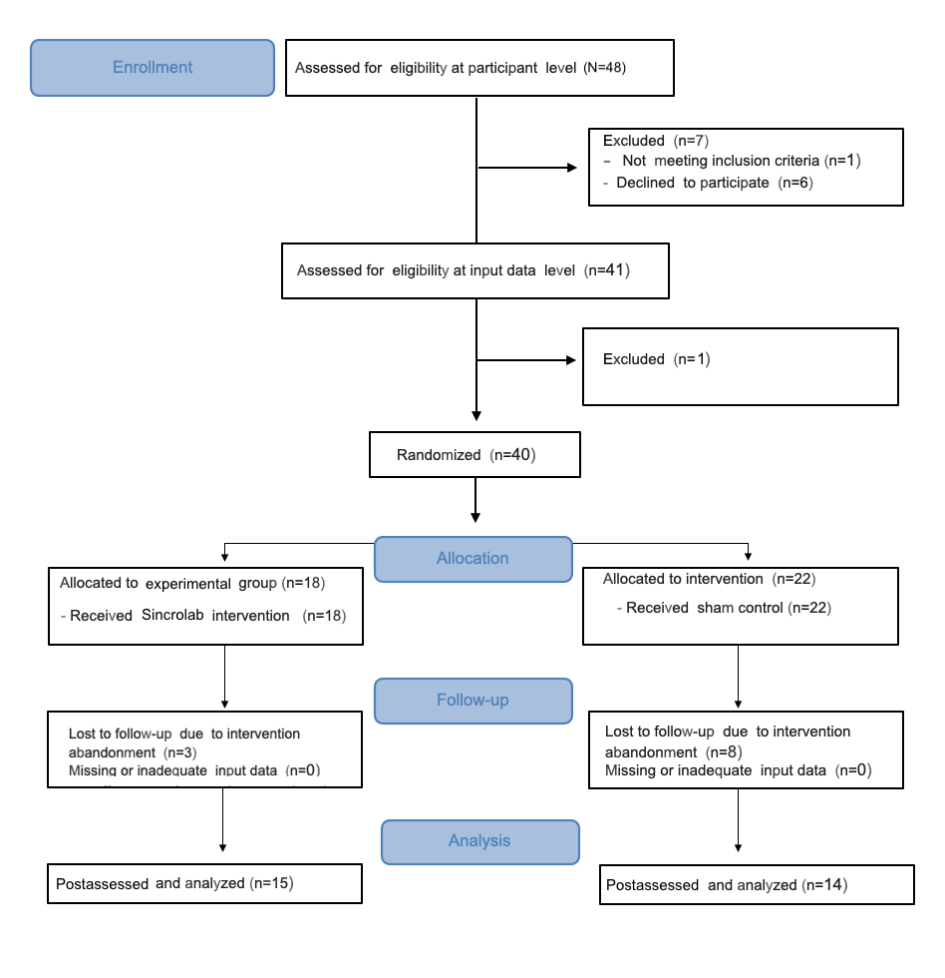
Consolidated Standards of Reporting Trials flow diagram.

From the initial pool of 41 volunteers, 40 were randomly allocated into 1 of the 2 trial conditions (experimental or control). Of these, 28% (n=11) dropped out during the intervention period (control=8, experimental=3). One participant did not the meet inclusion criterion of stopping ADHD medication prior to treatment. The Consolidated Standards of Reporting Trials (CONSORT) 2010 flow diagram is presented in Figure 1.

From the final sample of 29 participants, 20 were taking pharmacological interventions (experimental=9, control=11), 7 participants were taking nonpharmacological interventions such as psychological interventions (experimental= 4, control=3), and 4 were taking both (experimental=2, control=2). The pharmacological interventions were based on methylphenidate (Concerta; n=7), methylphenidate (Equasym; n=7), methylphenidate hydrochloride (Medikinet; n=2), lisdexamphetamine (Elvanse; n=2), and methylphenidate hydrochloride (Rubifen; n=2).

### Experimental Design

This was single-center, parallel, single-blind, randomized controlled trial that examined a pediatric population (8-11 years) diagnosed with ADHD of combined presentation. It was conceptualized as a proof-of-concept study intended to assess the preliminary efficacy of a digital, videogame-like, cognitive stimulation therapy, as well as its safety and engagement. Proof-of-concept trials are useful in the framework of novel drugs and devices, so knowledge regarding their administration (eg, dosing, user instructions) may be acquired in small samples in order to develop larger clinical trials [51,52].

### Digital Intervention

#### Experimental Condition

KAD_SCL_01 games are designed to work on different cognitive processes with an increase of the cognitive load following evidence that the brain’s reconfiguration networks seem to be fixed by this type of training routine [43-46,53]. The 14 games which compose the KAD_SCL_01 cognitive intervention are described in Multimedia Appendix 1. The game level is adapted based on a case-based reasoning algorithm. This algorithm and the human-AI interaction are described in Multimedia Appendix 2.

#### Control Condition

Participants received a sham intervention composed of 3 videogames which were not specifically designed to improve cognitive performance [54]. The specifications are described in Multimedia Appendix 3. The sham intervention tasks are accessible through Kongregate open-access platform (Kongragate Inc).

### Main Outcome Measure and Magnetoencephalography

#### Main Outcome Measure

The main outcome measure of this study was the change in score found in the commission score from Conners CPT (CPT-III) between both groups’ differences (pre- and postintervention) [55]. Commissions in CPT-III as a measure of inhibitory control was chosen as main outcome measure due to its use as an efficacy intervention measure in several previous studies about the methylphenidate effect in ADHD [56].

#### Magnetoencephalography

Neurophysiological data were acquired using a whole-head Elekta-Neuromag MEG system with 306 channels (Elekta AB) at the Center for Biomedical Technology (Madrid, Spain). MEG data were collected at a sampling frequency of 1000 Hz and online band-pass filtered between 0.1 Hz and 330 Hz.

Head shape was defined relative to 3 anatomical locations (nasion and bilateral preauricular points) using a 3D digitizer (Fastrak), and head motion was tracked through 4 head-position indicator coils attached to the scalp. Eye movements were monitored by a vertical electrooculogram assembly composed of a pair of bipolar electrodes.

### Other Cognitive Outcome and Clinical Outcome Measures

The secondary cognitive outcome, aimed at measuring other several aspects of cognitive processing, and clinical questionnaires on ADHD behavioral symptoms and executive functioning in daily activities are included in Multimedia Appendix 4.

### Safety and Compliance

Intervention safety was assessed through adverse events. Potential adverse events were monitored and recorded during the intervention period. Intervention dropouts were also recorded in order to assess compliance with intervention protocol.

### Study Procedure

The study procedure occurred in 4 stages: recruitment and screening, preintervention assessment (visit 1), at-home intervention, and postintervention assessment (visit 2). Recruitment and screening were carried out as described in the Participants section. The details of the Al are described in Multimedia Appendix 5.

Preintervention and postintervention assessments were performed at the Center for Biomedical Technology, at the Technical University of Madrid. Assessments were carried out by a blinded Sincrolab researcher (JB) who only knew the number associated with the participant. Assessments including neuropsychological batteries and MEG recordings were administered in the same order as reported here. Questionnaires for the clinical outcome measures were filled out by the legal guardians. The cognitive assessment lasted for approximately 60 minutes. The resting-state MEG was also recorded during visits 1 and 2. The order in which participants received both was counterbalanced.

The intervention allocation was created by a nonblinded Sincrolab researcher (RM) and performed with a simple randomization function, with a ratio of 1:1 and an allocation probability of 0.50. Intervention allocation was performed once the eligibility criteria were met, according to the 2010 CONSORT statement [57].

The intervention was scheduled for 12 weeks, with 3 sessions (15-20 minutes each) per week in both groups. The whole intervention period was telematically monitored. Both the KAD_SCL_01 and sham control platforms allowed for a daily checking of performed sessions for a nonblinded Sincrolab researcher (RM). The number of weekly intervention sessions performed by the participants was monitored to ensure compliance with the 12-week intervention protocol. Safety and adequacy (the number of games played and the consecutive extreme punctuations of 0 or 100 in the performance, which could reflect an issue in the calibration of the AI outputs) were also assessed. Legal guardians were contacted by study staff in order to report any adverse event.

Right after the at-home intervention period was over, participants who achieved at least 80% completion of intervention sessions (28 alongside the 12 prescribed weeks) were appointed for postintervention assessment with same characteristics as the preintervention one. After the postintervention assessment, the participants who were allocated in the control arm were offered training with the KAD_SCL_01 for 12 weeks.

### Statistical Analyses

Data analysis in this proof-of-concept randomized trial followed a per-protocol approach [58]. A per-protocol population was defined as any participant who had been randomly allocated to 1 of the 2 conditions (experimental or control), complied with at least an 80% completion of scheduled sessions (28 of 36), and had received the postintervention assessment.

#### Statistical Analyses of Cognitive Outcome Measures

Descriptive statistics of average, distribution shape, and scatter were calculated. Standardized statistics of asymmetry and kurtosis were used to assess the normality assumptions of each distribution. These standardized statistics are calculated by dividing the statistic between its SE.

Next, cognitive outcome measures which did not deviate from normality were adjusted to mixed-effects models. Each model was adjusted with a random intercept and fixed slope (due to the number of repeated measures). An unstructured covariance matrix (Sigma) was estimated for the random effect factor. Robust restricted maximum likelihood was chosen as the estimation method of preference due to its robustness with small samples and its capability to estimate an unbiased parameter matrix in the presence of missing values. A stepwise method was used for age as a demographic covariable in the main outcome’s mixed model as a method applied to explicative models.

As the commission score from CPT-III was set as main outcome measure, only 1 comparison was performed (1 dependent variable). Therefore, no correction for multiplicity was applied. Regarding the rest of the cognitive outcome measures, every *P* value under significance α value of .05 was taken as statistically significant due to the exploratory nature of this pilot study. Still, *P* values were corrected for multiple comparisons under a Bonferroni correction within a statistical family. The outcome measures from the different cognitive processes (ie, visuospatial working memory) were treated as independent statistical families for Bonferroni adjustments.

Effect sizes greater than 0.4 (considered as the minimum practical effect size [59] in the experimental condition but not in the control condition) were highlighted. Likewise, for the main outcome, the predictive positive value (PPV) was estimated, as the small sample size could have led to overestimation of the effect size. Due to the novelty of this type of training methodology, a priori effect size and unspecified prestudy odds (*R*=0.5) were used in order to estimate the PPV.

Respondent analysis was also performed over the main cognitive outcome measure (commission score on CPT-III) in order to study the proportion of participants per intervention arm who achieved a pre-post difference of at least 0.64 SD, according to other literature [56]. Moreover, with consideration to this a priori effect and because the estimated sample size could not be achieved, post hoc power analysis for the mixed model’s interaction component was carried out with 200 simulations, and PPV was computed following the procedure in Button et al [60].

#### Statistical Analyses of Clinical Outcome Measures

Clinical outcome measures were standardized according to Behavior Rating Inventory of Executive Function (BRIEF) and Evaluación del Trastorno por Deficit de Atención e Hiperactividad (EDAH) standardized scores (*T* scores). Paired-samples *t* tests were performed over each outcome measure and in each intervention group. Respondent analysis was also performed on the EDAH outcome measures by counting the proportion of participants who reached the cutoff point of pathology set by the interpretation of the EDAH manual for each condition. BRIEF and EDAH were treated as independent statistical families for Bonferroni adjustments.

#### Magnetoencephalography Signal Preprocessing and Statistical Analyses

With the intention of facilitating this paper’s interpretation, signal preprocessing analyses are detailed in Multimedia Appendix 6.

Regarding the statistical analyses of the MEG preprocessed signal, the aim of this study was the detection of any robust correlation between power ratio values derived from the clusters of nodes localized in certain brain regions and CPT’s commission ratio (CPT commission postintervention or CPT commission preintervention). The goal of this methodology included the extraction of any neurophysiological markers whose dynamic could be associated with the evolution of the inhibition-control performance. Such analysis relied on network-based statistics [61,62]. Clusters were built according to a criterion of spatial and frequency adjacency. Each cluster consisted of several adjacent nodes, which systematically showed a significant partial correlation (with age as the covariate) in at least 4 consecutive frequency steps (a 1-Hz interval) between their corresponding power ratio values and CPT ratio (Spearman correlation coefficient *P* value <.05). Importantly, all nodes within a cluster needed to show the same sign of the correlation coefficient for the cluster to be considered a functional unit. Only clusters involving at least 1% of the nodes (ie, a minimum of 12 nodes) in each frequency step were considered. Cluster-mass statistics were assessed through the sum of the Spearman ρ values across all nodes and significant frequency steps.

To control for multiple comparisons, the entire analysis pipeline was then repeated 5000 times, with the correspondence between power ratio estimates and CPT ratio being shuffled across participants. At each repetition, the maximum statistic of the surrogate clusters (in absolute value) was kept, creating a maximal null distribution that would ensure control of the familywise error rate at the cluster level. Cluster-mass statistics on each cluster in the original data set were compared with the same measure in the randomized data. The network-based statistics *P* value represented the proportion of the permutation distribution with cluster-mass statistic values greater or equal to the cluster-mass statistic value of the original data.

Power ratio values were averaged across all nodes and frequencies that belonged to the cluster. This average was considered to be the representative MEG marker value for that cluster and further participated in subsequent correlation analyses. Therefore, the statistics presented in the results section was derived from the correlation between the averaged power ratio value of each significant cluster and the corresponding CPT ratio for each participant. As already mentioned, correlations were first performed within the whole sample. In a second step, correlations between the average power ratio and the CPT commission ratio scores were performed independently for both intervention conditions within the sample (experimental and control). Statistical analyses were carried out using MATLAB R2020b (Mathworks Inc).

### Sample Size Justification

A priori sample size was estimated to detect a standardized mean difference of 0.64 SD in the commission score from the CPT-III [56], with a significance level of α=.05 and a power of 0.8 (1-β=.8). The calculation procedure followed the sample size estimation for a 2-tailed, 2-samples mean difference with a correction factor for repeated measures [63]. The total sample size required was 56, but the actual sample was 29. Nevertheless, sample sizes of between 10 and 15 participants per condition are well-supported in similar literature [64-66].

The last enrolled participant ended study procedures in February 2020. With the COVID-19 crisis and the consequences in Spain (since March 2020), the study sponsor and principal investigator (FM) decided to stop the recruitment procedures due to difficulties and in order to assure protocol compliance in 2020. Therefore, assuming the exploratory nature of this pilot randomized trial, it was decided that the statistical analysis plan be applied to the presented sample.

## Results

### Demographic and Baseline Characteristics

Baseline demographics and other characteristics in each group, as well as the between-group comparison, are shown in Table 1. No significant differences were found between groups.

**Table 1:** Demographic characteristics in the experimental and control conditions.

Characteristic	Experimental group, (%)^a^ (N=15)	Control group, (%)^a^ (N=14)	*t* or *X*^2^	*P* value
Age (years)	9.2 (1.21)	9.71 (1.33)	1.09	.27^b^
Males	13 (44.8)	12 (41.4)	0.005	.94^c^
Using medication	9 (31)	11 (37.9)	1.17	.28^c^
Receiving psychological treatment	4 (13.8)	3 (10,3)	0.11	.74^c^

^a^The characteristic of age is expressed as mean (SD).

^b^*P* values are from a *t* test (between-participant, 2-tailed).

^c^*P* values are from a chi-squared test (2-tailed).

### Safety and Compliance

Three adverse events were reported by legal guardians during the at-home intervention period (Multimedia Appendix 7). Dropout (n=11) details are shown in Multimedia Appendix 8.

### Main Outcome

**Figure 2:**
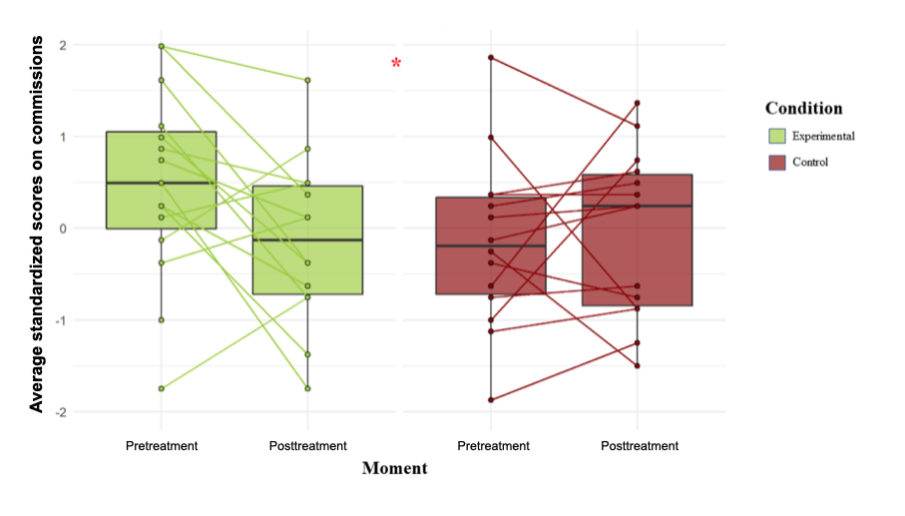
Main efficacy outcome: individual and average change in commission errors from Conners continuous performance test per condition.

Descriptive statistics for the main outcome measure in each condition at each study period are shown in Table 2. The evolution trend (pre-post training) of each participant and the distribution of each group is shown in Figure 2. No statistically significant difference was found between the conditions (experimental and control) in the preintervention (baseline) measures (t_27_=1.72; *P*=.10). Critical ratios for skewness indicated no deviations in skewness or kurtosis in the normal distribution.

**Table 2:** Descriptive statistics for main outcome measure commission score on Conners continuous performance test (CPT-III).

Descriptive statistic	Treatment group		Control group	
	Pretreatment	Posttreatment	Pretreatment	Posttreatment
Mean (SD)	53.87 (8.37)	47.80 (8.21)	48.79 (7.53)	49.64 (7.32)
Asymmetry	–0.37	–0.17	0.28	–0.09
Kurtosis	–0.61	–0.76	–0.33	–1.52
CR^a^ asymmetry	–0.32	–0.15	0.23	–0.08
CR kurtosis	–0.27	–0.34	–0.14	–0.66

^a^CR: critical ratio.

Mixed-effects models for main outcome measured with and without interaction effects were estimated with the robust restricted maximum likelihood procedure. The stepwise introduction of the condition-period interaction effect significantly improved the model adjustment (*X*^2^_1_=4.596; *P*=.03). Standardized mean difference (β estimator) for the condition-period interaction effect in the final model (Figure 2) was statistically different from 0 (β=.86; SE 0.39; t_27_=2.21; *P*=.04). The standardized mean differences (β estimators) for model comparison (baseline model to final model) are shown in Multimedia Appendix 9. Comparison criteria (Akaike information criterion and Bayesian information criterion) between the models, in addition to model performance statistics (*R*^2^ and adjusted *R*^2^), are also reported in Multimedia Appendix 9. The graphical diagnosis for the final model with interaction effect is shown in Multimedia Appendix 10.

Pre-post standardized mean differences per condition were calculated as Hedges g statistic for effect size. A large pre-post standardized mean difference (g>|0.4|) was found in the experimental group (g=–0.62), but not in the control group (g=0.1). A high PPV (PPV=0.81) was found to be related with the pre-post standardized mean difference.

Respondent analysis for the main outcome measure shows that 53.33% (8/15) of the experimental participants (KAD_SCL_01 intervention) achieved the a priori clinically meaningful effect: an improvement of at least 0.64 standardized points. In the control arm, this percentage was just 21.42% (3/14). More details about respondent analysis are shown in Multimedia Appendix 11.

A post hoc power analysis yielded a statistical power of 43% (1 – β=0.43) for the detection of the condition-period interaction effect and a PPV of 0.81. A priori effect size was used to simulate post hoc power rather than observed effect size.

### Magnetoencephalography Outcomes

**Figure 3:**
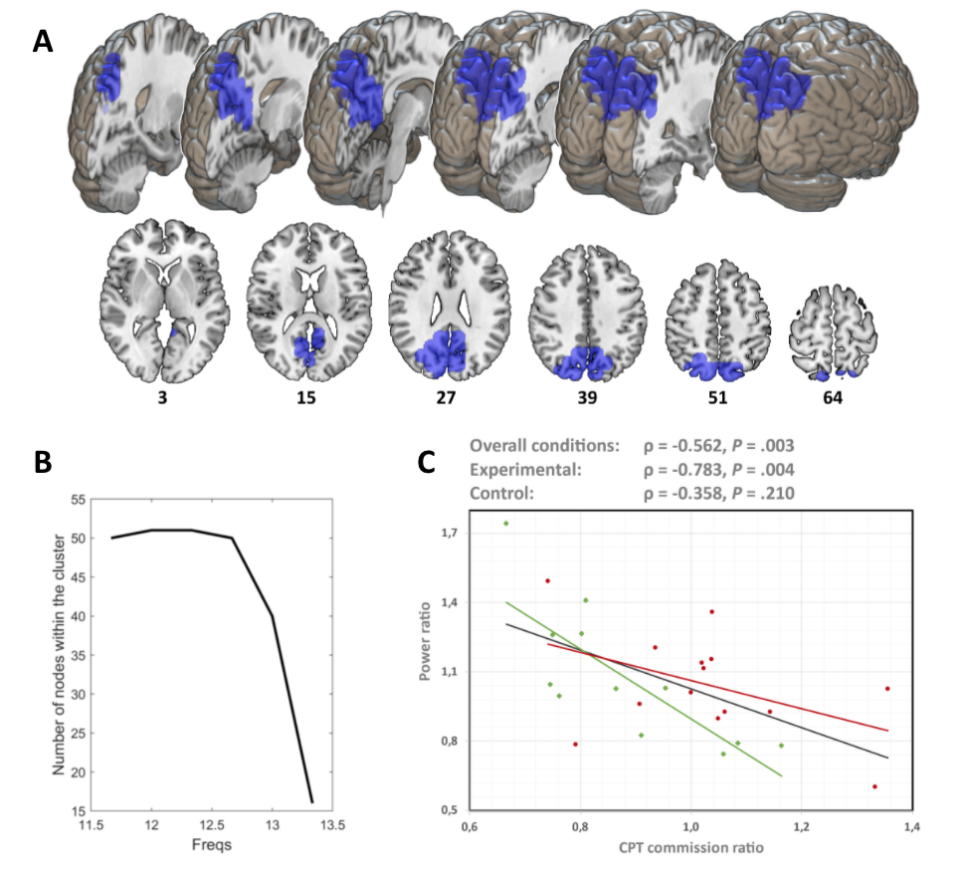
Brain region whose magnetoencephalography alpha power (11.67-13.33 Hz) was found significantly correlated with CPT commission ratio. (A) Brain regions within the significant cluster (depicted in blue). (B) Evolution of the cluster size through the different frequency steps (maximum size at 11.75 Hz). (C) Scatter plot showing the Spearman correlation coefficient between the cluster’s average power ratio and CPT commission ratio and each subgroup of the sample. CPT: continuous performance test; Freqs: frequency steps.

A significant cluster (*P*=.04) was found in the frequency interval (11.67-13.33 Hz) mainly comprising the posterior regions of the brain (see Figure 3A and Table 3).

The power ratio in all frequencies of this interval negatively correlated with the CPT ratio across the whole sample (ρ=–0.562; *P*=.003). The maximum cluster size was found at 12-12.33 Hz (51 nodes). The cluster size oscillated between a minimum of 50 nodes at the beginning of the frequency range and 16 at the end of that frequency range (see Figure 3B). Furthermore, 12 Hz showed the highest average correlation coefficient value across all nodes of the cluster ρ= –0.547).

The correlation between the CPT commission ratio and the power ratio (11.67-13.33 Hz) in the interval within the cluster generated in the previous step remained significant for the experimental group (ρ=–0.783; *P*=.004; Figure 3C) but not for the control group (ρ=–0.358; *P*=.21; Figure 3C).

**Table 3:** The automated anatomical labeling atlas ROIs^a^ that were partially captured by the significant cluster.^b^

ROI	Portion of ROI occupied, n/N (%)^c^
Left precuneus	11/28 (39.29)
Right precuneus	8/21 (38.10)
Left cuneus	7/11 63.64)
Right cuneus	7/13 (53.85)
Right superior parietal gyrus	6/18 (33.33)
Left cingulate gyrus, posterior part	3/5 (60.00)
Right superior occipital lobe	3/10 (30.00)
Left superior parietal gyrus	2/16 (12.50)
Right cingulate gyrus, posterior part	1/4 (25.00)
Left calcarine fissure and surrounding cortex	1/20 (5.00)
Left superior occipital lobe	1/11 (9.09)
Right middle occipital lobe	1/17 (5.88)

^a^ROI: region of interest.

^b^Regions of interest are from the Anatomical Labeling Atlas that are part of the significant cluster where the continuous performance test commission ratio correlates with power in the alpha band.

^c^N is the number of magnetoencephalography sources in our head model that are contained within the ROI volume; n indicates how many sources, among the corresponding N, are enclosed within the significant cluster; and % is the percentage of each ROI that was captured by that cluster.

### Other Cognitive and Clinical Outcomes

Descriptive analysis for each secondary outcome measure is shown in Supplementary Material (Multimedia Appendix 12). No statistically significant differences were found between conditions (experimental and control) in preintervention measurement.

The mixed-effects model analysis was performed the for main outcome measure. Only, the backward span score (from the Corsi block-tapping test) as a dependent variable (*X*^2^_1_=4.64; *P*=.03) was significant. The standardized mean difference for the condition–moment interaction effect (β estimator) in the final model was statistically different from 0 (β =–.84; SE 0.38, t_27_=–2.24; *P*=.03). Multimedia Appendix 13 shows the graphical representation of the average pre- and postintervention standardized scores for each intervention group (experimental and control) in this outcome measure. Standardized mean differences and CIs for cognitive secondary outcomes are shown in Multimedia Appendix 14, as classified by the cognitive process each measures (inhibitory control, cognitive flexibility, working memory, short-term memory, attention, speed processing, and verbal fluency).

Effect sizes of g>0.4 in the experimental group but not the control group were found in 19 cognitive secondary outcome measures, plus the main outcome. In contrast, only 1 cognitive secondary outcome measure showed a greater effect size (g>0.4) in the control group compared to the experimental one. See Multimedia Appendix 15 for the complete analysis.

The results in the parent version of the BRIEF questionnaire showed statistically significant pre-post mean differences, favoring the KAD_SCL_01 intervention participants in shifting score (t_14_=2.32; *P*=.03), working memory score (t_14_= 2.43, *P*=.02), behavioral composite index (t_14_=2.62, *P*=.02), and general executive composite index (t_14_= 2.7, *P*=.01). No significant differences in the sham intervention group were found. The experimental arm (KAD_SCL_01 intervention) showed statistically significant pre-post mean differences in all EDAH measures (hyperactivity score *P*=.05), inattention score (*P*=.001), behavior disorder score (*P*=.001), and global score (*P*=.001). The control arm also showed statistically significant pre-post mean differences in inattention score (*P*=.001), hyperactivity + inattention, and global score (*P*=.002), but not in hyperactivity or behavior disorder score. Respondent analysis, descriptive analysis, *t* statistics, *P* values, CIs, and respondent percentage per score in the EDAH scale are detailed in Multimedia Appendix 16.

No statistically significant differences were found between conditions (experimental and control) in the preintervention measurement either in the BRIEF or the EDAH outcome measures.

## Discussion

Empirical evidence points suggests that cognitive stimulation based on progressive workload increments leads to improvements in cognitive performance [38-42,67], along with beneficial regulation of cortical activity patterns [43-46,53]. 

The results in our study indicate that cognitive intervention triggers significant improvements in inhibitory control in child and adolescent patients with ADHD as measured by Conners CPT-III. Moreover, this improvement in inhibitory control seems to be similar to that found in pharmacological studies on the effectiveness of methylphenidate [68]. Meanwhile, the effect size of our study (g=0.62) is consistent with that found in the meta-analysis by Losier et al [56] on the effectiveness of the drugs used in ADHD. Therefore the digital treatment proposed in the present study could be a therapeutic option complementary to the pharmacological route.

Despite there being no significant differences between the groups in the measure of previous treatment (*P*=.09), the possible differences between both groups could be producing a type I error or false-positive result. However, as observed in Figure 2, within the range of 1 to –1 SD, 7 of the 10 patients who received the KAD_SCL_01 treatment show an improvement in their performance (70%). On the contrary, 6 of 9 participants belonging to the control group, in the same range, show worse scores in the postintervention measure.

Although several studies have reported that digital cognitive exercises do not show effects superior to those found in other commercial video games not intended for therapeutic uses [37,69,70], these findings, like those reported by Davis et al [41] and Kollins et al [42], seem to indicate that adaptive digital training, built on a proven empirical basis, could be effective for the treatment of ADHD.

The relationship between alpha-band power and performance in tasks involving attentional and inhibitory control processes has been published in recent publications [14-16,71,72]. In order to clarify the relationship between the changes in inhibitory control and the possible changes in alpha band power—given its association with performance in inhibitory control tasks [14,15]—we completed a MEG registry of the participants of both groups. The results seem to indicate that there is a direct relationship (ρ=–0.56; *P*=.003) between the improvement in inhibitory control and the alpha-band power in the posterior brain areas. These changes in the power of brain oscillations appear to be associated with brain plasticity processes [73]**,** as well as changes in the dynamics of neuromodulators such as dopamine [74] that are affected in these patients. This relationship remained significant when the experimental group (ρ= –0.78; *P*=.004) was analyzed separately, but this was not the case with the control group (ρ= –0.35; *P*=.21). This suggests that the improvements produced in the experimental group are strongly associated with the previously mentioned plasticity and neuromodulation phenomena.

Although this was intended as a power study, we believe our results are relevant to the functional connectivity literature due to the participation of the precuneus and posterior cingulate cortex in the main cluster examined of this paper. These 2 regions of interest conform to the posterior part of the DMN. Consequently, the alpha-power increment linked with the CPT decrement may be associated, under our interpretation, with an improvement in the functional integrity of the DMN. The decrease in alpha-band power in regions of the DMN could be mediating the impairments present in ADHD in the functional connectivity of this network [75-77] and its neurocognitive correlate [34,35,78]. This effect seems to be due to frequency band having special importance in the communication between the regions of this network [33]. In this regard, the data from Sonuga-Barke and Castellanos [79] seems to indicate that the decrease in connectivity between the regions of the DMN generates interference in the task-oriented network, producing impairments in the performance of patients with ADHD [79]. From this perspective, this digital cognitive stimulation intervention, based on progressive workload increases governed by CBR algorithms, might be effective for the treatment of ADHD.

One of the secondary objectives of the study was to measure the effectiveness of the intervention on a set of cognitive processes that are usually part of the ADHD deficits. The results indicate that cognitive intervention triggers improvements in visuospatial working memory total score. Moreover, practical minimum effect size (g> 0.4) was observed in visuospatial working memory span and in visuospatial working memory total score, while numeric working memory total score or numeric working memory span seemed to be not be affected by the treatment. These results are similar to those found by another digital study, which based its training on empirical principles [41]. In our study, no significant differences were found in other measures. However, these results might be due to a type II or false-negative error, since small sample sizes frequently generate this type of error [80]. Indeed, we found a practical minimum effect size in 12 of the 42 secondary outcome indices, which is a possible indicator of treatment efficacy. In contrast, the control group showed equivalent effects in just 1of the 42 secondary measures (see Multimedia Appendix 15 for detailed information).

Finally, in this study, the parent version of the BRIEF questionnaire was used to obtain a measure of executive functions in everyday life. The results indicate that cognitive training triggers significant changes in flexibility, working memory, and the composite indices of both behavior and executive functions. These changes appear to be similar to the effects of methylphenidate-based pharmacological treatments [81] and treatments administered by clinical professionals for executive functions [82].

In the ADHD rating scale, in the overall rating, 60% (9/15) of the participants who underwent cognitive training exceeded the cutoff point (<30), compared to 21% (3/14) who worked with commercial video games, which seems to indicate that this type of cognitive training may have positive effects on the behavioral impact of the disorder. 

In conclusion, this study reports the preliminary results of a digital cognitive stimulation intervention in a population with ADHD. The results suggest that such treatment is effective at improving inhibitory control and visuospatial working memory in patients with ADHD. Moreover, this improvement was observed in the executive measures of daily life and was associated with a reduction of symptoms.

The main limitation of the study relates to the small size of the sample (N=29) compared to the a priori calculated sample size (N=56). Consequently, the statistical power was lower than the one desired a priori. Therefore, these results must be interpreted as the first evidence of a digital treatment using CBR algorithms, and more extensive studies are needed to confirm the findings of this proof-of-concept study.

## Appendix

Multimedia Appendix 1 Dynamics, integrated cognitive processes, and hierarchical or multilevel structure of Sincrolab’s games.

Multimedia Appendix 2 Case-based reasoning explanation and human-artificial intelligence interaction.

Multimedia Appendix 3 Description of sham control intervention.

Multimedia Appendix 4 Cognitive and clinical secondary outcome measures.

Multimedia Appendix 5 Study procedure diagram.

Multimedia Appendix 6 Magnetoencephalography signal preprocessing procedure.

Multimedia Appendix 7 Adverse events.

Multimedia Appendix 8 Dropouts' details.

Multimedia Appendix 9 Comparison between performance and standardized mean differences (beta estimators) for each main outcome model.

Multimedia Appendix 10 Graphical diagnosis for main outcome's final model with interaction effect.

Multimedia Appendix 11 Comparison of individual performance and clinical effect.

Multimedia Appendix 12 Descriptive statistics for secondary outcome measures.

Multimedia Appendix 13 Efficacy outcome in visuospatial working memory: mean change in backward span score from Corsi block-tapping test. Significant differences were found for condition × moment interaction effect in this cognitive measure (β=–.84; SE=0.38; <italic>t</italic><sub>27</sub>=–2.24; <italic>P</italic>=.03).

Multimedia Appendix 14 Standardized mean differences for interaction effects in secondary outcome measures.

Multimedia Appendix 15 Pre-post standardized mean differences (Hedges g) per treatment group.

Multimedia Appendix 16 Descriptives, <italic>t</italic> statistics, <italic>P</italic> values, CIs, and respondent percentage per score in the Evaluación del Trastorno por Deficit de Atención e Hiperactividad scale.

Multimedia Appendix 17 CONSORT-eHEALTH checklist (V 1.6.1).

## Abbreviations

ADHD: attention deficit hyperactivity disorder
ADHD-C: attention deficit hyperactivity disorder combined type
AI: artificial intelligence
BRIEF: Behavior Rating Inventory of Executive Function
CBR: case-based reasoning
CONSORT: Consolidated Standards of Reporting Trials
CPT: continuous performance test
CPT-III: Conner continuous performance test
DMN: default mode network
DSM-5: Diagnostic and Statistical Manual of Mental Disorders, Fifth Edition
EDAH: Evaluación del Trastorno por Deficit de Atención e Hiperactividad
MEG: magnetoencephalography
PPV: predictive positive value
