# Supplementary material for: Electrophysiological Brain Changes Associated With Cognitive Improvement in a Pediatric Attention Deficit Hyperactivity Disorder Digital Artificial Intelligence-Driven Intervention: Randomized Controlled Trial
Source: J Med Internet Res. 2021 Nov 26;23(11):e25466. doi: 10.2196/25466 (PMC8665400; doi:10.2196/25466)

Annex S2. The Sincrolab S.L. Artificial Intelligence (AI) engine, built via the machine learning paradigm and technique known as Case-Based Reasoning (CBR) and the human-AI interaction

Case-based reasoning

The problem we need to resolve regarding the Sincrolab S.L. AI engine is: What level of difficulty of the task is optimal for an individual, within a given context? This context is made up of variables that include the patient's levels of cognitive impairment, age and current performance, whose characterisation and use will be revealed in later phases of this document.

Sincrolab S.L.'s AI engine makes use of knowledge from similar past cases to solve the current problem. These cases are based on different individuals presenting the same context.

The memory modules of the Sincrolab S.L. AI engine contain information about the contextual aspects of individuals and the level of task performance or resolution. Its hierarchical structure is well defined within the work cycle – Case selection.

CBR allows for the following question to be solved: 'what level of difficulty of the task is optimal for an individual, within a given context?', as a result of the following characteristics:

- As it is not necessary to work with an explicit domain, we can deal with the individualised cognitive profile. This allows us to analyse its special features.
- It allows the number of stored cased to be increased. This increases the system's knowledge with the special features of the case in terms of its context and performance.

The principles that regulate CBR allow the Sincrolab S.L. AI engine's behaviour to solve the problem posed. Likewise, it allows it to act in a way which is analogous to that of an expert therapist. The principles of CBR and its application at Sincrolab are as follows:

- Regularity: If the approach to a task was appropriate for a patient who presented the same characteristics in terms of context and results as the current patient, their performance faced with the same task should be the same or similar.
- Typicity: Faced with a new patient within a given context, it is likely that another patient with the same or similar characteristics has been treated in the past.
- Consistency: Small differences in the new patient, in relation to the most similar patients, should produce slight changes in the interpretation and proposed solution.
- Adaptability: Because the differences in the new patient are small, changes in the proposed solution should also be small and easy to produce.

The CRB work cycle is applied and defined as follows:

Case selection

Given a new patient, the system carries out a search for cases stored in the knowledge system. As a result, there are multiple types of information collection. The knowledge and selection of cases is carried out as follows:

A) The most similar cases are searched for in the AGE parameter, for which a type of knowledge based on measurements is used.

B) From the selection taken from the previous point, the most similar cases are searched for via the cognitive profile parameter, for which a type of knowledge based on vocabulary and measurements is used.

The cognitive profile parameter is a parameter composed of multiple characteristics. These features are organised hierarchically into domains and subdomains. These include:

- Attention [Sustained attention, Selective attention, Alternating attention, Divided attention, Visual divided attention, Auditory divided attention, processing speed].
- Memory [Working memory, Updating, Phonological working memory, Numerical working memory, Visuospatial working memory, Short-term memory, Visual short-term memory, Numerical short-term memory, Verbal short-term memory].
- Executive functions [Flexibility, Planning, Inhibition control, Attention control, Categorization, Visual-motor coordination, Problem solving, Anticipation, Generation of alternative responses, Strategy, Supervision, Decision making, Use of feedback].
- Visuospatial skills [Visuospatial perception, Spatial localisation, Visuospatial orientation].
- Mathematics [Calculus, Mathematical logical reasoning].
- Language [Phonological path, Lexical path, Access to the lexicon].

Associated with each of these subdomains is a natural number whose value is between 0 (no knowledge) - 6 (maximum performance) that determines the level of involvement of each of the patients in each of the domains.

Searching for cases in the knowledge base is carried out in two steps:

a) Similarity between subdomains with knowledge/no knowledge. Type of knowledge: vocabulary.
b) Similarity between values of the knowledge selected in the previous step. Type of knowledge: measurement.

C) From the selection taken from the previous point, cases with the greatest similarity in the difficulty of the task that the new case has completed in its most recent performance are selected. The level of difficulty of the task is defined hierarchically in its structure.

The upper level: these are the task's game modes. These determine the number and level of cognitive processes involved in the task.

The lower level: these are the positive real values that determine each of the parameters that define the task in the given mode.

D) From the selection taken from the previous point, cases with an equal or similar performance in the task to that which the new case has obtained in its last performance are selected. This is a positive real value between 0 and 1.

E) From the selection taken from the previous point, a solution to the following problem is provided: 'What level of difficulty of the task is optimal for an individual, within a given context?' For this, the configuration of the task that was presented to the selected cases is adopted for the following task.

Adaptation: structural adaptation

An average is taken from the parameters that define the task for the selected cases from the knowledge base. The aim is to obtain a measurement that assumes a similar value, although slightly different for the new case. The principles that govern this adaptation are those of consistency and adaptability.

Solution assessment

For the problem posed: 'What level of difficulty of the task is optimal for an individual, within a given context?' The solution is: that produced by an average performance, understanding this as an IC (0.4 - 0.7).

Updating the knowledge base

The new case is stored in the knowledge base.

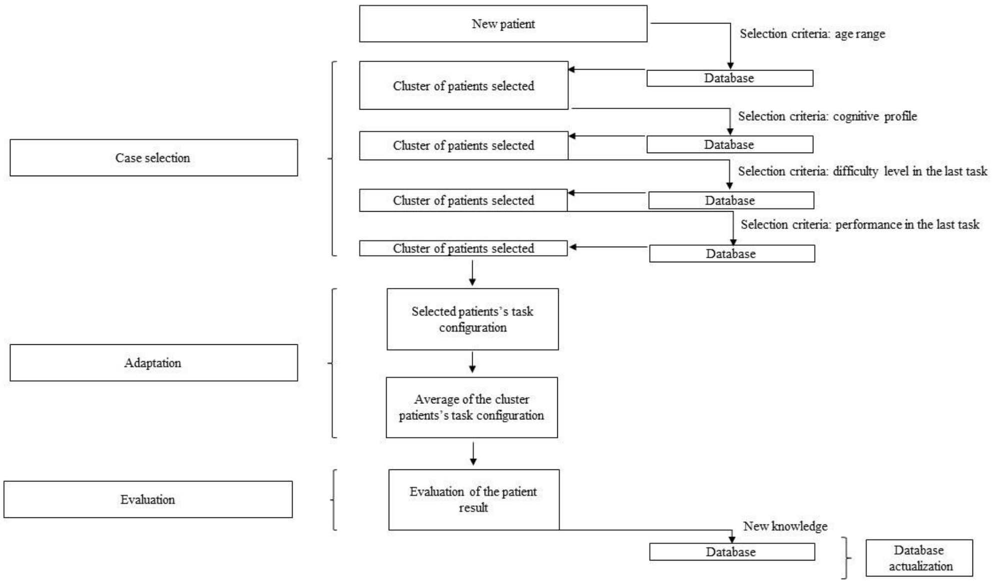

Human-AI interaction

Interaction human-artificial intelligence is only required when the supervisor therapist registers a new patient in the SINCROLAB's environment. In this registration process, patient's demographic (age, sex, handness) and cognitive data are introduced by the therapist and this will determine the comparative group (reference group) for this concrete patient. Likewise, these demographic and cognitive data will define, within an interval, the difficulty parameters and the number of cognitive processes for each game which will compose the first intervention session.

Next, the registration process followed by the supervisor therapist in order to register a new patient in SINCROLAB's environment is introduced step by step. This process is performed in the SINCROLAB's professional application. This application is access-limited for mental health professionals, so no patient is able to access to his/her own clinical data.

1. First, demographic information about the patient (age, sex and handness) is introduced. Also, in this first step, the patient is identified within SINCROLAB's environment with a code (no personal ID information is required) and his/her personal access' keys are generated (username and password). Although sex and handness are collected, both variables are not used as predictive or explicative factors in this version of de AI

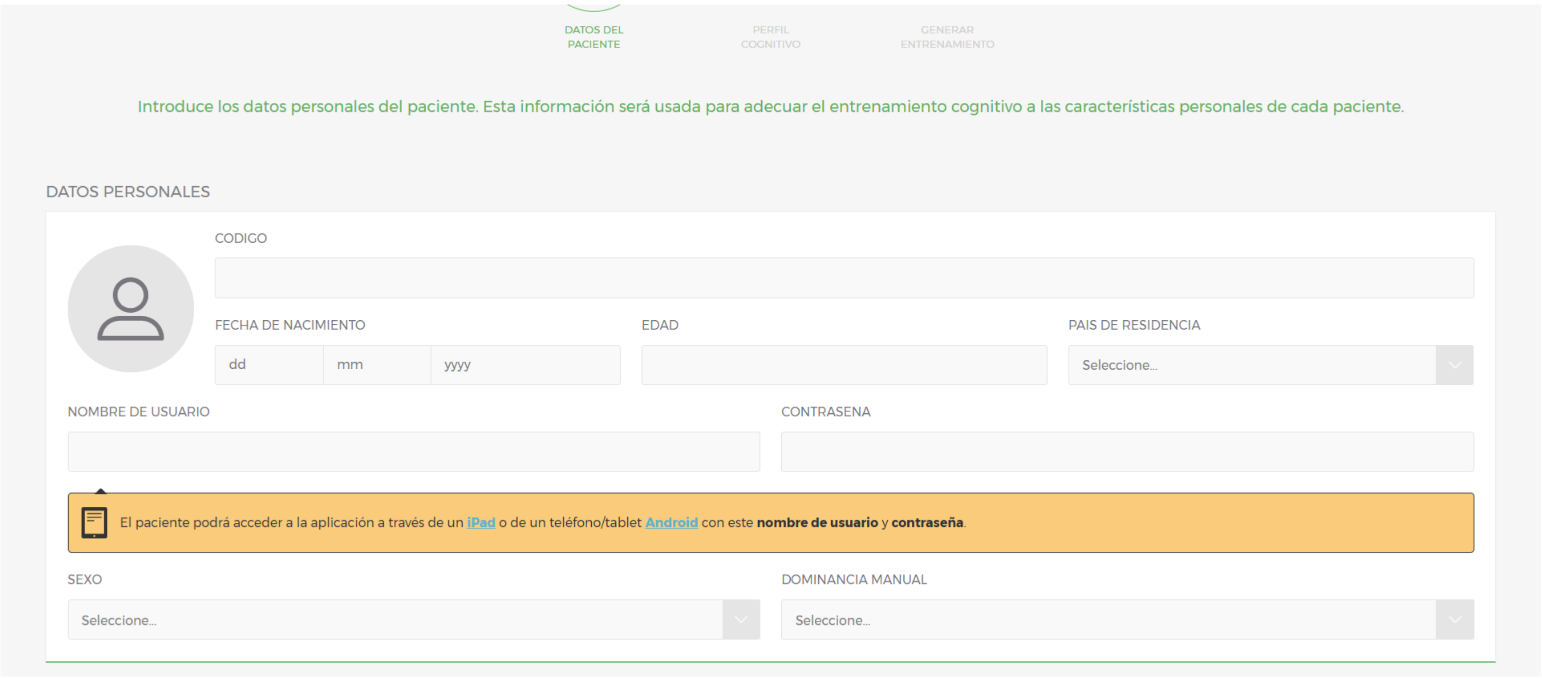

2. In the second step, patient's cognitive profile is defined. In this study, this profile is defined through "Detailed diagnostic" option as "Attention deficit and hyperactivity disorder of combined presentation". This step, along with the age, defines the patient's initial reference group. This initial reference group allows to assign the initial level of difficulty for the first intervention session.

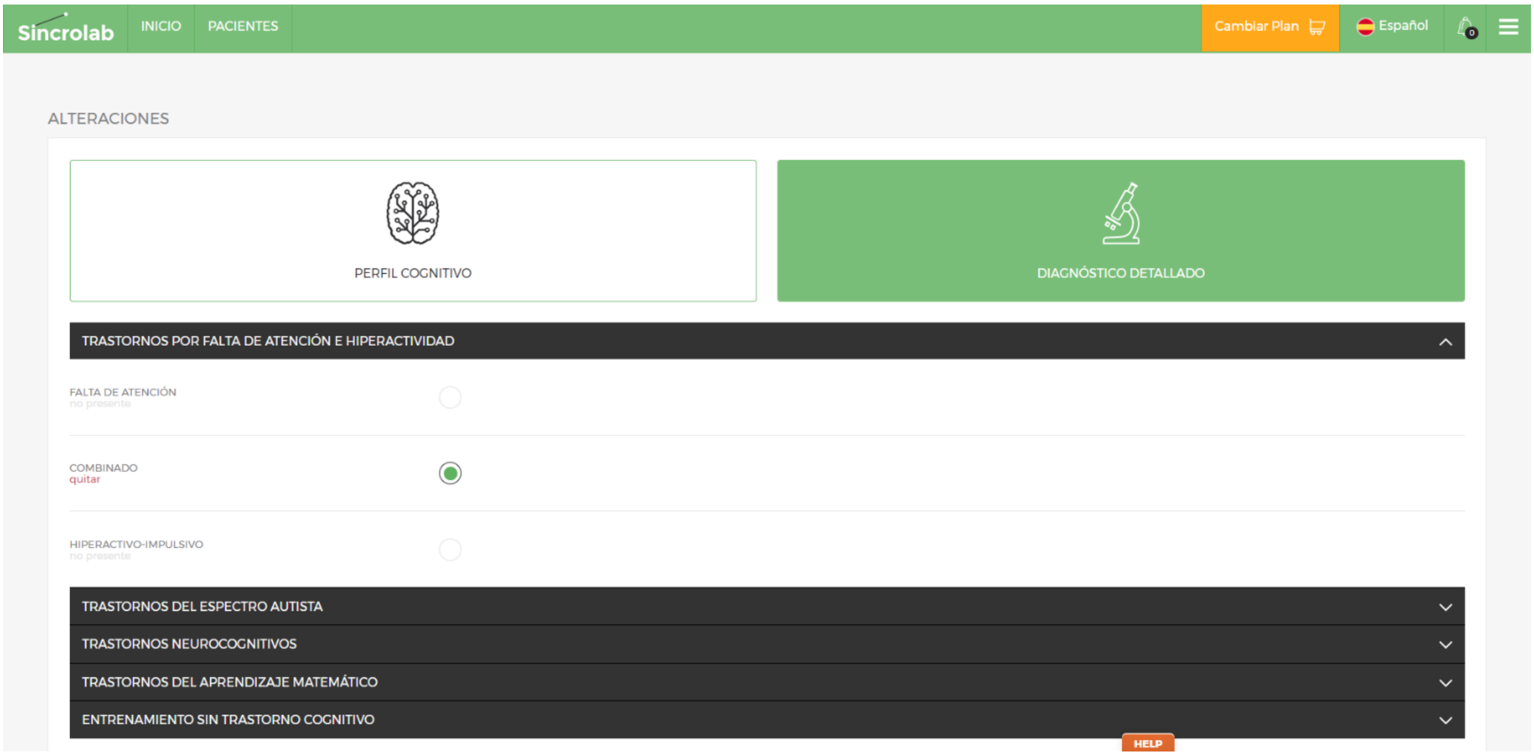

3. In the third and last step, the time (in minutes) and the number of games for each intervention session is established. In this study, these parameters are set to 15 minutes and 3 games per intervention session.

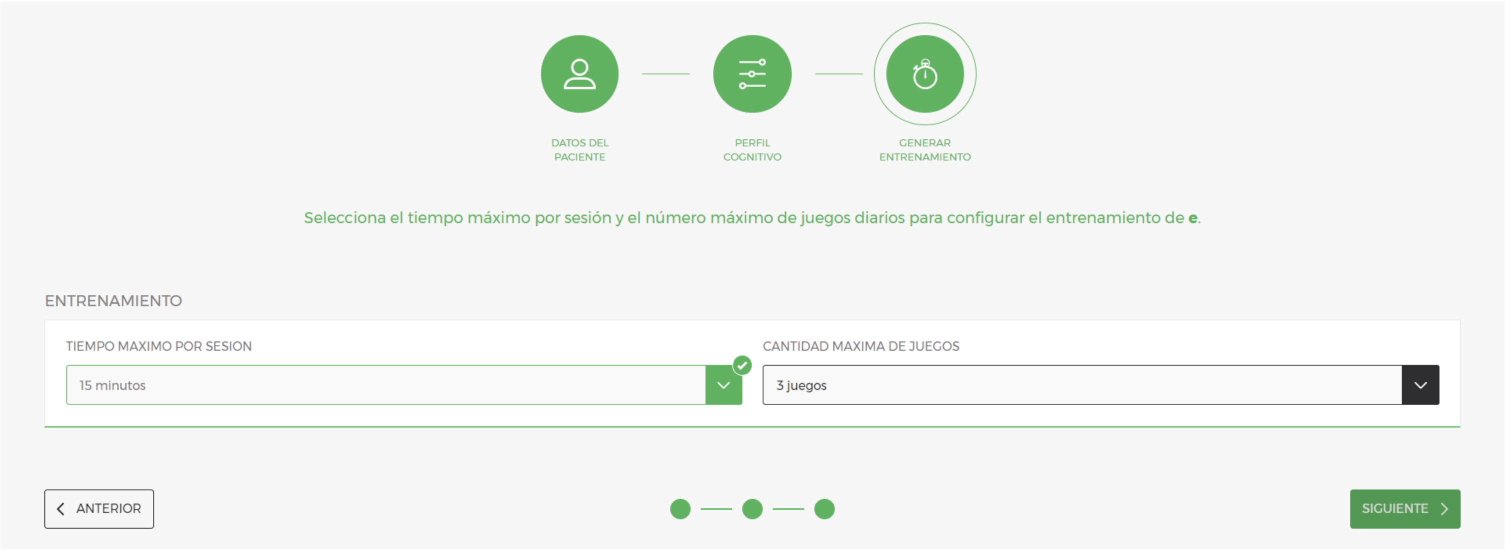

Supplement: Multimedia Appendix 2 [file jmir_v23i11e25466_app2.pdf]
