# Supplementary material for: Electrophysiological Brain Changes Associated With Cognitive Improvement in a Pediatric Attention Deficit Hyperactivity Disorder Digital Artificial Intelligence-Driven Intervention: Randomized Controlled Trial
Source: J Med Internet Res. 2021 Nov 26;23(11):e25466. doi: 10.2196/25466 (PMC8665400; doi:10.2196/25466)

Annex S3. Sham Intervention

The videogames were Knightmare Tower, Bloons Super Monkey and Super Staker 2. Knightmare Tower is a runner-like videogame in which the player has to ascend to the top of a tower while avoiding enemies and traps. Bloons Super Monkey is a videogame, similar to the classic Space Invaders, in which the player has to defeat enemies and obstacles by moving left or right. Last, Super Stacker 2 is a puzzle-like videogame in which the player has to locate a certain number of geometrical pieces in order to keep them balanced.

Knightmare Tower

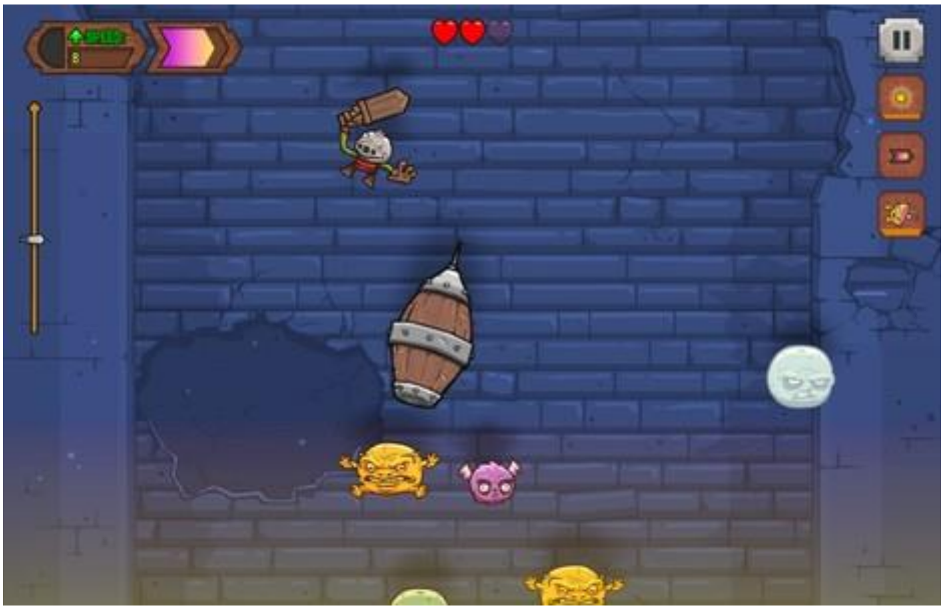

Bloons Super Monkey

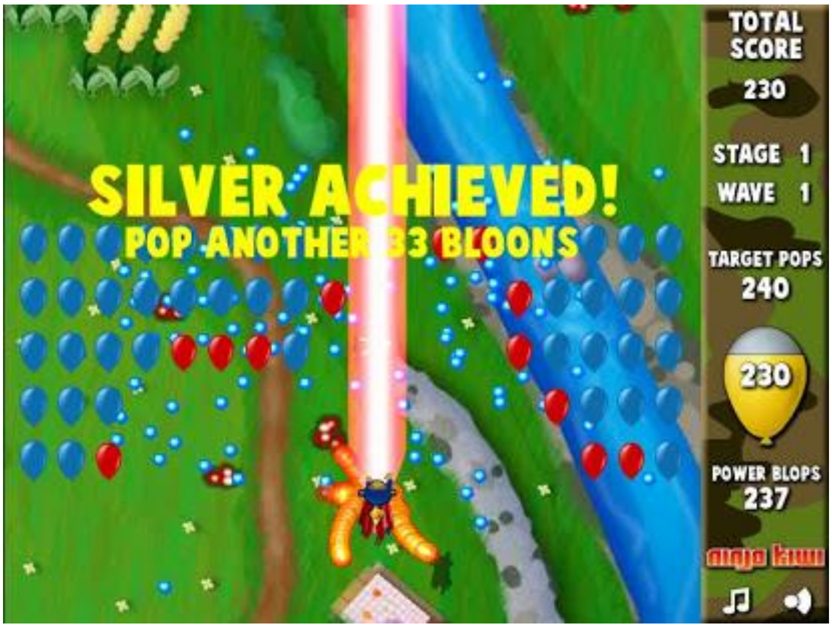

Super Staker 2

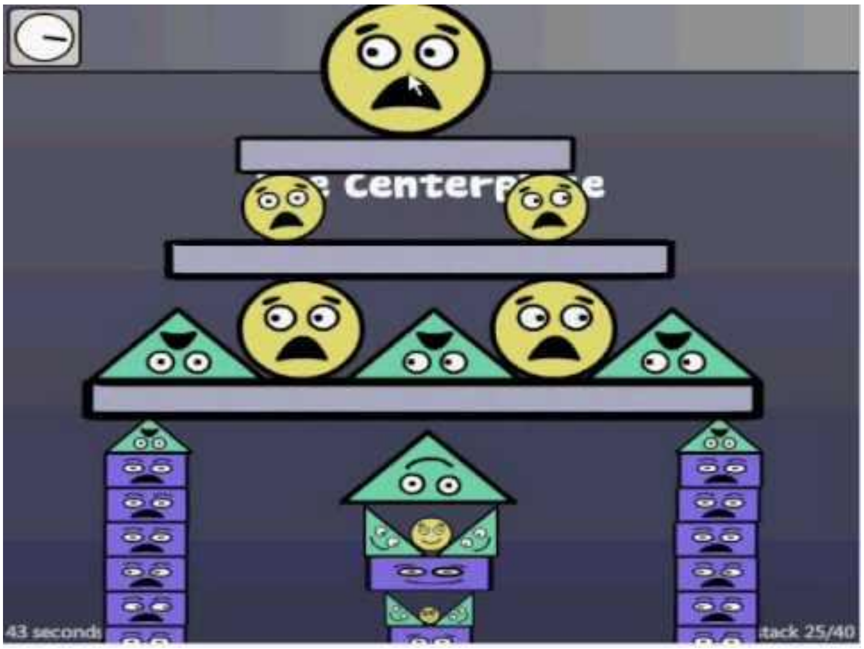

Supplement: Multimedia Appendix 3 [file jmir_v23i11e25466_app3.pdf]
