# Supplementary material for: Electrophysiological Brain Changes Associated With Cognitive Improvement in a Pediatric Attention Deficit Hyperactivity Disorder Digital Artificial Intelligence-Driven Intervention: Randomized Controlled Trial
Source: J Med Internet Res. 2021 Nov 26;23(11):e25466. doi: 10.2196/25466 (PMC8665400; doi:10.2196/25466)
Supplement: Multimedia Appendix 4 [file jmir_v23i11e25466_app4.pdf]

Annex S4. Cognitive and clinical secondary outcome measures

Cognitive secondary outcome measures

Conners' Continuous Performance Test-III (CPT-III): CPT-III is a computerized continuous performance task which measures several aspects of attention and executive functioning. In this version, stimuli (14 letters; letter X is the target and the others are the distractors) are presented individually in the laptop screen. Participants have to press the space bar each time a distractor appears in the screen and inhibit this response when a target is shown. The task is composed by 360 trials, grouped by 8 blocks with variable inter-stimuli interval (ms) for each block. In addition to main outcome measure (commission errors), omission and perseveration errors, detectability index, hit reaction time (HRT), standard deviation of HRT (HRT SD), response variability and HRT Inter-Stimulus Interval (ISI) change (HRT-ISI) were taken.

Developmental Neuropsychological Assessment-II (NEPSY-II): NEPSY-II is a standardized neuropsychological battery, widely used in order to assess a vast range of cognitive processes, along the neurodevelopment. Psychometric properties have been published within the Spanish adaptation, including reliability, internal consistency, test-retest stability and internal and external validity. The following subtests were administered.

- *Auditory Attention and Cognitive Flexibility Test.* This test is based on an auditory detection task in which participants listen to a sequence of words from a recorded audio tape. Participants have to point the target in a piece of paper every time they listen to it. Targets are colors, and they are presented in a piece of paper with four colored circles (red, green, blue and black). In *Auditory Attention*, target word is *"RED"* (*"ROJO"* in Spanish). Every time the participants listen to the word *"ROJO"* from the audio tape, they have to point the red circle in the piece of paper. In *Cognitive Flexibility* condition, there are three target: *"RED", "GREEN"* and *"BLUE"* (*"ROJO", "VERDE"* and *"AZUL"* in Spanish). When they listen to the word "*RED",* participants have to point the green circle, and, on the contrary, when they listen to the word *"GREEN"*, they have to point the red circle. In addition, when participants listen to the word *"BLUE"*, they have to point the blue circle. From Auditory Attention Test and Flexibility Test, number of correct answers and number of commissions, omission and inhibition errors were taken.

- *Verbal Fluency Test.* This is a classical verbal fluency task in which participants have to verbally declare as many words as they are able to, following a certain condition, in 1 minute per condition. There are four conditions in total. These conditions are classified in *Semantic* and *Phonological*. In *Semantic* section, conditions are words which belong to the semantic categories of *Animals* and *Food.* In *Phonological* section, conditions are words which start with the letter *P* and with the letter *M.* Number of correct answers per section (Semantic and Phonological) is the selected outcome measure.

- *Card Classification Test.* In this test, a pack of eight cards with pictographic representations of animals in their natural habitats are given to the participant. The objective is to perform sequential classifications for these cards in two groups (4 cards per group) according with a *classification rule* which must be inferred by participant. There are 10 possible classification rules (e.g the size of the animals, the weather, the color of the card). Number of correct answers, repeated errors, *other classification* errors (classifications according with other rules rather than the possible ten ones) and total errors were taken.

- *Inhibition Test.* Inhibition Test from NEPSY-II is a Stroop-based task in which a sequence of 40 black and white geometrical shapes (circles and squares) is presented in paper. Participants have to identify each item from the sequence by saying in out loud *"CIRCLE"* or "*SQUARE"* (*"CÍRCULO"* or *"CUADRADO"* in Spanish) as faster as they are able to, under three different conditions. First, as the items appear in the sequence; second, exchanging the shapes (circles as squares and squares as circles); and third, naming the black figures as they are shown and the white figures exchanging its identification (white circles as squares and white squares as circles). From this test, response time, number of errors and number of self-corrected errors were obtained.

<u>Wechsler Intelligence Scales for Children-IV (WISC-IV):</u> WISC-IV is a cognitive standardized battery which aims to measure different aspects of cognitive processing. Psychometric properties have been published within the Spanish adaptation, including reliability, internal consistency, test-retest stability and internal and external validity. The following subtests were administered.

- *Digit Span Test.* In this test, participants are asked to verbally repeat a sequence of digits, whose length goes increasing by one integer after each pair of trials, starting from a sequence of two digits. Two conditions are administered: *forward* (forward repetition of the sequence of digits) and *backward* (backward repetition of the sequence of digits). The administration of each condition ends after three consecutive wrong answers. This test is designed in order to measure verbal short-term and working memory. Number of correct answers in each condition (*Total Forward* and *Total Backward*) were computed. *Span scores* (length of the last sequence successfully repeated) for each condition *(Forward Digit Span* and *Backward Digit Span)* were also taken.
- *Symbol Search Test.* Symbol Search is a test in which participants have to determine if one of the two target symbols is hold in a sequence of symbols. If there is at least one target between the sequence, they have to mark *"YES"* in the item's answer box; else, they have to mark "*NO"*. Participants have to solve as many items as they are able to in two minutes. This test intends to measure rapid processing and visuospatial attention. Number of correct items, number of errors and total processed items are take taken.
- *Digit Symbol Substitution Test.* In this test, the nine integers from 1 to 9 are paired to different symbols. These matches compound the substitution guide. The test consists of a sequence of 126 integer numbers (from 1 to 9) in which participants have to write below each integer its paired symbol following the substitution guide. They have two minutes to fulfill as many integers as they are able to. Number of correct answers, number of errors and total processed integers are take taken.

<u>Corsi Block Tapping Test from Wechsler Non-Verbal Scales (WNV):</u> This test is a compound of the WNV battery and intends to measure spatial working memory. Stimuli are 9 equal blue cubes (or blocks) on a wood table. As in the Digit Span Test, evaluator performs a sequence by pressing the blocks in an order determined by test instructions and participants have to repeat this sequence (each sequence composes one trial). The length of the sequence increases by one block after each pair of trials. Two conditions are administered: *forward* (forward repetition of the block sequence) and *backward* (backward repetition of the block sequence). The administration of each condition ends after three consecutive wrong answers. From this test, number of correct answers in each condition (*Total Forward* and *Total Backward*) were computed. *Span scores* (length of the last sequence successfully repeated) for each condition *(Forward Digit Span* and *Backward Digit Span)* were also taken.

Clinical secondary outcome measures

Clinical questionnaires about ADHD behavioral symptoms and executive functioning in daily activities compose the clinical secondary outcome measures section.

Behaviour Rating Inventory of Executive Function, Parent Version (BRIEF): BRIEF is an 86-item questionnaire which assess executive functioning in daily-life. It rates different behaviors which are related with executive function disorders from 1 to 3 according with its frequency and it is widely administered in ADHD assessment. BRIEF has been adapted to Spanish infant population with reliable psychometric properties and criterion validity with EDAH questionnaire From BRIEF questionnaire (Parent Form), scores of Inhibition, Shifting, Working Memory, Behavioral and Global scales were computed and taken.

Evaluation of Attention Deficit and Hyperactivity Disorder (EDAH): This questionnaire is a Spanish adaptation of the Conners' Rating Scales with reliable psychometric properties. It is aimed to measure pediatric ADHD symptomatology (inattention, hyperactivity, behavioral disorders). From EDAH questionnaire, scores of Hyperactivity, Attention Deficit, Behavioral Disorder and Global subscales were computed and taken.
