# Supplementary figures and images for: Electrophysiological Brain Changes Associated With Cognitive Improvement in a Pediatric Attention Deficit Hyperactivity Disorder Digital Artificial Intelligence-Driven Intervention: Randomized Controlled Trial
Source: J Med Internet Res. 2021 Nov 26;23(11):e25466. doi: 10.2196/25466 (PMC8665400; doi:10.2196/25466)

**Figure S1. Study Procedure Diagram**

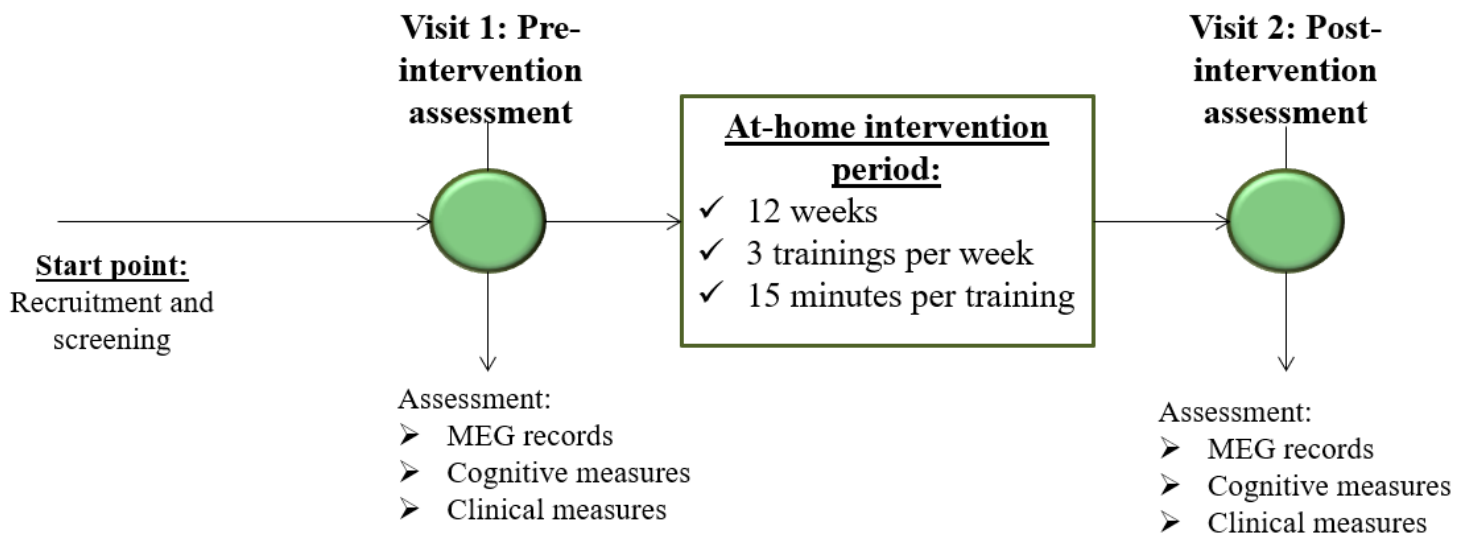

Supplement: Multimedia Appendix 5 [file jmir_v23i11e25466_app5.pdf]

**Figure S2. Graphical diagnosis for final model (main outcome) with interaction effect**

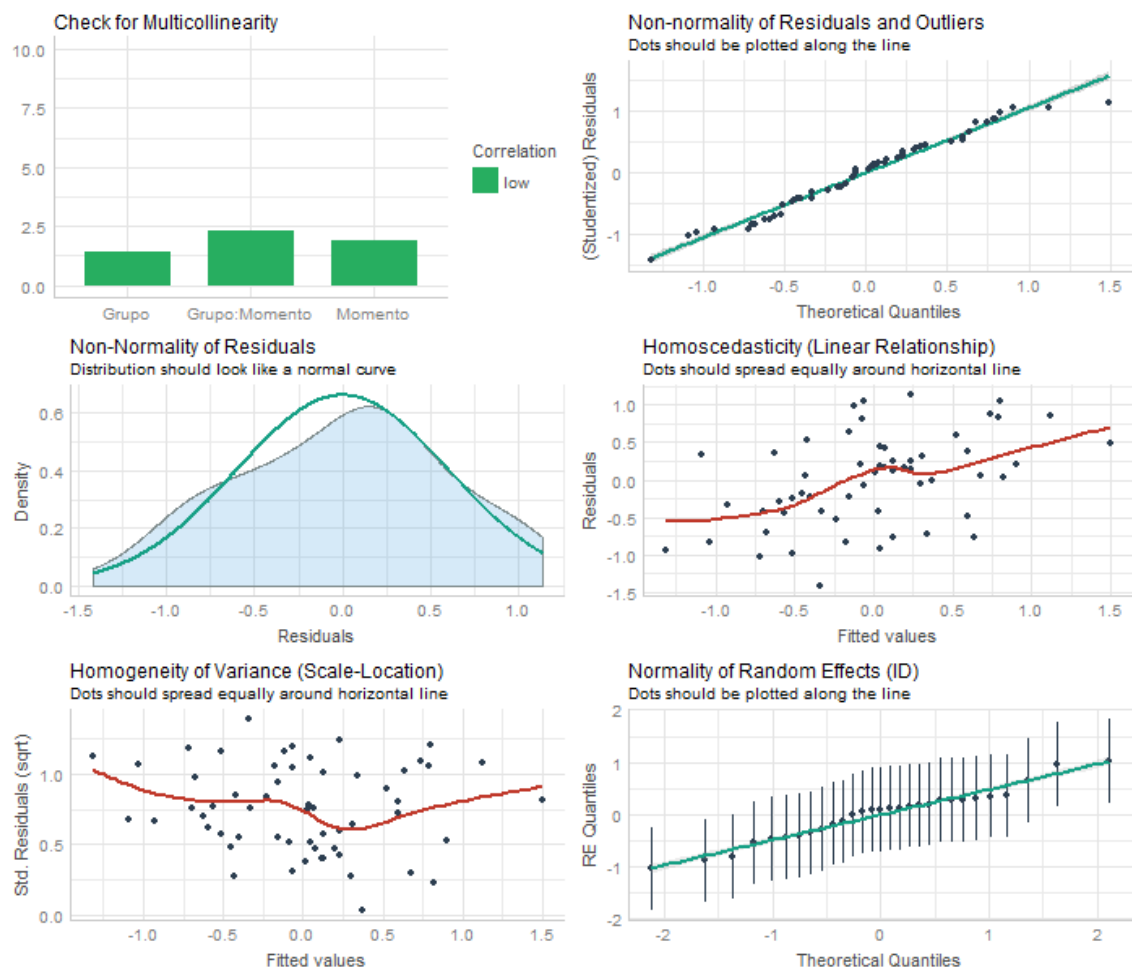

Supplement: Multimedia Appendix 10 [file jmir_v23i11e25466_app10.pdf]
