# Supplementary material for: Electrophysiological Brain Changes Associated With Cognitive Improvement in a Pediatric Attention Deficit Hyperactivity Disorder Digital Artificial Intelligence-Driven Intervention: Randomized Controlled Trial
Source: J Med Internet Res. 2021 Nov 26;23(11):e25466. doi: 10.2196/25466 (PMC8665400; doi:10.2196/25466)
Supplement: Multimedia Appendix 6 [file jmir_v23i11e25466_app6.pdf]

Annex S6 Magnetoencephalography signal pre-processing

Raw recording data was first introduced to Maxfilter software (v 2.2, correlation threshold = 0.9, time window = 10 seconds) to remove external noise using the temporal extension of the signal space separation method with movement compensation. Then, magnetometers data was automatically examined to detect ocular, muscle, and jump artifacts using *Brainstorm* software, which were visually confirmed by an MEG expert. The remaining artifact-free data was sectioned into four-second segments. Afterwards, signal-space projection analysis-based procedure (SSP) was applied to remove heart magnetic field artifacts and EOG components. Only those recordings with at least 20 clean segments (80 seconds of brain activity) were utilized in subsequent analyses. MEG clean time series were band-pass filtered (0.5 second padding) between 2 and 45Hz.

As no individual anatomy was available, source reconstruction was carried using a template head model. The head model consisted of a single layer representing the inner skull interface, generated from the union of tissues gray matter, white matter and cerebrospinal fluid in the MNI brain. The source model was defined as a regular grid of 10 mm spacing defined in MNI space, and only those sources falling in an area defined as cortical in the Automated Anatomical Labeling atlas was considered, resulting in 1202 source positions. The scalp of the MNI template was linearly transformed to the individual head shape using an affine transformation generated with an iterative algorithm, and the same transformation was applied to the head and source models. The leadfield was calculated using a single shell model. Sources time series were reconstructed using a Linearly Constrained Minimum Variance beamformer, using the trial-average covariance matrix and a regularization factor of 5% of the average sensor power.

Power spectrum of each grid's node was computed by means of Fast Fourier Transform using Hanning tapers with 0.25 Hz smoothing. For each node, relative power was calculated by normalizing by total power over the 1.5- to 45-Hz range. Since power in the alpha band was the focus of the study, only frequency steps (25 in total) within the interval [8-14Hz] were considered for the analyses. The source template with 2459 nodes in a 10 mm spacing grid was segmented into 78 regions of the Automated Anatomical Labeling atlas, excluding the cerebellum, basal ganglia, thalamus and olfactory cortices. Those 78 regions of interest included 1202 of the original 2459 nodes. Trials were

averaged across subjects ending up with a source-reconstructed power matrix of 1202 nodes x 25 frequency steps x 24 participants. Finally, the power-ratio (post-condition/pre-condition) was calculated to assess the change between the two conditions of the follow-up. This final power-ratio matrix was employed in the correlation analysis.
