# Supplementary material for: Electrophysiological Brain Changes Associated With Cognitive Improvement in a Pediatric Attention Deficit Hyperactivity Disorder Digital Artificial Intelligence-Driven Intervention: Randomized Controlled Trial
Source: J Med Internet Res. 2021 Nov 26;23(11):e25466. doi: 10.2196/25466 (PMC8665400; doi:10.2196/25466)
Supplement: Multimedia Appendix 7 [file jmir_v23i11e25466_app7.pdf]

Table S1. Adverse events

Condition	AEs	Severity	Relation to the intervention	Did it prevent to perform intervention session as usual?
Experimental	Headache	Slight	Unlikely	No
Control	Influenza	Mild	Not related	Yes
	Familiar issue	Mild	Not related	Yes
