# Supplementary material for: Electrophysiological Brain Changes Associated With Cognitive Improvement in a Pediatric Attention Deficit Hyperactivity Disorder Digital Artificial Intelligence-Driven Intervention: Randomized Controlled Trial
Source: J Med Internet Res. 2021 Nov 26;23(11):e25466. doi: 10.2196/25466 (PMC8665400; doi:10.2196/25466)
Supplement: Multimedia Appendix 8 [file jmir_v23i11e25466_app8.pdf]

Table S2. Dropout details

	Control	Experimental
Never started	3	0
Complications unrelated to treatment (family and medical)	1	1
Technical problems (No internet access for holidays)	1	2
Engagement	3	0
