# Supplementary material for: Electrophysiological Brain Changes Associated With Cognitive Improvement in a Pediatric Attention Deficit Hyperactivity Disorder Digital Artificial Intelligence-Driven Intervention: Randomized Controlled Trial
Source: J Med Internet Res. 2021 Nov 26;23(11):e25466. doi: 10.2196/25466 (PMC8665400; doi:10.2196/25466)
Supplement: Multimedia Appendix 9 [file jmir_v23i11e25466_app9.pdf]

Table S3. Comparison between performance and standardized mean differences (beta estimators) for each main outcome model

Fixed effects	Initial model	Final model	Age covariate
	Beta [95% CI]	Beta [95% CI]	Beta [95% CI]
(Intercept)	0.27<br>[-0.20, 0.73]	0.47<br>[-0.02, 0.97]	1.46<br>[-0.88, 3.80]
Condition	-0.20<br>[-0.81, 0.40]	-0.63<br>[-1.35, 0.08]	-0.58<br>[-1.31, 0.15]
Moment	-0.34<br>[-0.75, 0.07]	-0.76 **<br>[-1.29, -0.22]	-0.76 **<br>[-1.29, -0.22]
Condition x Moment		0.86 *<br>[0.10, 1.63]	0.86 *<br>[0.10, 1.63]
Age			-0.11<br>[-0.36, 0.14]
AIC	171.05	168.45	172.04
BIC	181.35	180.82	186.46
R2 (fixed)	0.04	0.08	0.10
R2 (total)	0.40	0.48	0.49

** $p < 0.01$; * $p < 0.05$.
