# Supplementary material for: Electrophysiological Brain Changes Associated With Cognitive Improvement in a Pediatric Attention Deficit Hyperactivity Disorder Digital Artificial Intelligence-Driven Intervention: Randomized Controlled Trial
Source: J Med Internet Res. 2021 Nov 26;23(11):e25466. doi: 10.2196/25466 (PMC8665400; doi:10.2196/25466)
Supplement: Multimedia Appendix 11 [file jmir_v23i11e25466_app11.pdf]

Table S5. Comparison between individual performance improvements and clinical effect between intervention

	Performance[a]		Clínical effect[b]	
	Better after intervention	Not improved	Better after intervention	Worse after intervention
KAD_SCL_01®	73.33% (11/15)	26.67% (4/15)	53.33% (8/15)	6% (1/15)
Control	28.47% (4/14)	61.53% (10/14)	21.42% (3/14)	

[a]Pre-post standardized mean difference represents an improvement in performance

[b]Pre-post standardized mean difference represents an improvement of at least 0.64 SD
