# Supplementary material for: Electrophysiological Brain Changes Associated With Cognitive Improvement in a Pediatric Attention Deficit Hyperactivity Disorder Digital Artificial Intelligence-Driven Intervention: Randomized Controlled Trial
Source: J Med Internet Res. 2021 Nov 26;23(11):e25466. doi: 10.2196/25466 (PMC8665400; doi:10.2196/25466)
Supplement: Multimedia Appendix 12 [file jmir_v23i11e25466_app12.pdf]

Table S4. Descriptive statistics for secondary outcome measures

Neuropsychological instrument	Outcome measure	Treatment group						Control group					
		Pre-treatment			Post-treatment			Pre-treatment			Post-treatment		
		Mean (SD)	c.r Asymmetry	c.r Kurtosis	Mean (SD)	c.r Asymmetry	c.r Kurtosis	Mean (SD)	c.r Asymmetry	c.r Kurtosis	Mean (SD)	c.r Asymmetry	c.r Kurtosis
EDAH scales	Hyperactivity score	12.46 (3.39)	0.10	-1.07	8.66 (3.47)	-0.27	-0.81	11.78 (3.76)	-0.27	-0.64	8.5 (3.54)	0.06	-0.72
	Inattention score	13.33 (3.19)	-0.45	-0.41	9.26 (2.78)	0.41	-0.29	11.85 (4.07)	-0.29	-0.46	9.14 (3.57)	-0.04	-0.77
	Behavioural Disorders score	19.46 (7.94)	-0.02	-0.51	10.06 (6.11)	0.37	-0.39	17.92 (7.51)	-0.09	-0.67	10.78 (6.07)	-0.30	-0.57
BRIEF	Inhibition score	20.8 (3.85)	0.13	-1.42	20 (5.6)	-0.19	-0.61	22 (4.81)	-0.38	-0.46	20.35 (4.81)	0.22	-0.41
	Flexibility score	16.8 (2.70)	0.00	-0.87	15.6 (3.5)	0.20	-0.56	15.71 (3.53)	0.25	-0.60	16.14 (3.10)	0.09	-0.53
	Working Memory score	25.73 (2.84)	0.05	-1.40	24.46 (3.66)	-0.26	-0.66	24.14 (4.45)	-0.36	-0.49	23.42 (4.55)	-0.40	-0.38
Auditory Attention Test	Correct answers	27.28 (3.81)	-0.85	0.07	27.13 (2.79)	-0.58	-0.34	28 (3.23)	-0.54	0.28	28 (2.85)	-1.28	0.70
	Commissions	4.28 (2.64)	0.87	0.36	2.46 (1.50)	0.15	-0.51	3.35 (2.67)	1.09	0.22	2.35 (1.90)	0.22	-0.74
	Omissions	2.85 (3.39)	1.12	1.10	2.4 (2.82)	0.82	-0.12	2 (2.25)	0.66	-0.31	1.5 (2.76)	1.71	1.50
	Inhibition errors	2.07 (1.73)	0.19	-1.59	1.66 (1.71)	0.62	-0.34	2.42 (2.95)	1.09	0.28	1.07 (1.43)	0.87	-0.16
Cognitive Flexibility Test	Correct answers	27.33 (4.25)	0.00	-1.47	29.93(4.69)	-0.67	-0.28	27.35 (4.68)	-0.21	-0.42	30 (4.47)	-1.09	0.52
	Commissions	4.2 (2.93)	0.59	-1.02	1.8 (2.04)	0.99	0.19	6.14 (5.58)	0.83	-0.13	2.71 (2.94)	0.85	-0.22
	Omissions	5.93 (4.21)	0.38	-1.21	5.26 (4.62)	0.73	-0.24	5.64 (5.19)	0.83	-0.05	4.07 (4.82)	1.35	0.94
	Inhibition errors	3.53 (3.02)	0.97	0.04	1.6 (1.68)	0.88	0.22	4.78 (4.96)	1.01	0.15	1.78 (2.19)	0.89	-0.25
Digit Span Test	Forward correct answers	6.46 (0.91)	0.08	-1.01	6.66 (1.23)	0.70	-0.38	7.57 (2.44)	0.34	-0.49	7.5 (2.40)	0.97	0.56
	Forward span	4.46 (0.51)	0.10	-2.11	4.53 (0.74)	0.75	-0.35	5.28 (1.13)	0.29	-0.61	5.28 (1.32)	1.13	0.73
	Backward correct answers	5.93 (1.33)	0.68	-0.31	6.06 (0.96)	-0.10	-0.05	5.92 (1.59)	0.89	0.27	6.5 (1.99)	0.18	-0.30
	Backward span	3.26 (0.70)	0.69	0.40	3.53 (0.63)	0.56	-0.35	3.5 (1.01)	0.85	0.13	3.57 (1.01)	0.02	-0.56
Verbal Fluency Test (Semantic)	Correct answers	27.93 (8.68)	-0.11	-1.23	29.64 (5.37)	0.36	-0.59	28.07 (6.87)	-0.30	-0.12	25.71 (8.60)	-0.33	-0.06
Verbal Fluency Test (Phonological)	Correct answers	14.2 (6.13)	-0.04	-1.14	15.14 (5.46)	0.33	-0.49	14.92 (4.63)	-0.12	-0.55	15.5 (5.28)	-0.39	0.04
Inhibition Test	Errors	3.06 (4.63)	1.52	2.62	1.93 (1.98)	0.78	-0.16	4.42 (8.14)	1.84	1.73	3.5 (6.59)	2.02	2.21
	Self-corrected errors	4.06 (4.47)	1.12	0.83	3 (2.85)	0.71	-0.03	4.57 (4.46)	0.49	-0.41	4.57 (4.58)	0.29	-0.77
	Response time	24.6 (8.23)	0.16	-1.95	89.08 (48.06)	0.23	-0.38	56.66 (46.77)	0.33	-0.54	84 (40.63)	-0.30	-0.41
Corsi Block Tapping Test	Forward correct answers	6.6 (1.91)	-0.12	-1.69	7.6 (2.13)	0.17	-0.52	7.92 (2.75)	-0.06	-0.49	7.71 (2.67)	0.20	-0.37
	Forward span	4.86 (1.06)	0.21	-0.86	5.53 (1.45)	0.11	-0.44	5.5 (1.40)	-0.46	-0.46	5.21 (1.36)	0.26	-0.38
	Backward correct answers	5.8 (2.14)	-0.07	-1.29	7.28 (2.43)	0.22	-0.50	5.64 (1.94)	-0.58	-0.04	6.35 (2.46)	-0.49	-0.27
	Backward span	4.26 (1.16)	-0.20	-1.04	5.21 (1.57)	0.55	0.04	4.57 (1.34)	0.03	-0.37	4.35 (1.27)	-0.18	-0.56
Card Classification Test	Correct answers	3.2 (2.30)	0.40	-1.30	5.28 (2.58)	-0.08	-0.48	4.64 (2.61)	-0.28	-0.60	4.85 (2.45)	-0.26	-0.67
	Repeated answers	2.13 (1.50)	0.63	-0.71	1.28 (0.99)	0.28	-0.47	2.42 (1.65)	0.50	-0.22	2 (2.82)	1.41	0.85
	Unaccurate answers	1.8 (2.51)	1.92	4.64	1.07 (1.43)	0.87	-0.16	1.28 (1.71)	1.05	0.08	1.21 (1.62)	1.49	1.12
	Total errors	3.93 (3.53)	1.68	3.56	2.35 (1.82)	0.95	0.26	3.71(2.89)	0.68	-0.30	3.21 (4.07)	1.80	1.74
Symbol Search Test	Correct answers	22.66 (5.32)	-0.14	-0.51	23.4 (5.15)	0.30	-0.53	25.14 (4.89)	0.31	-0.42	26.28 (5.67)	0.46	-0.17
	Errors	3.13 (5.13)	1.98	4.80	1.26 (2.7)	2.40	3.08	2.71 (5.09)	1.93	1.97	3.28 (8.87)	2.45	3.14
	Processed stimuli	25.8 (6.65)	0.53	0.19	24.73 (4.38)	0.17	-0.44	27.85 (7.12)	0.64	-0.16	29.57 (10.40)	1.44	1.07
Digit Symbol Substitution Test	Correct answers	35.13 (8.04)	-0.60	-1.07	35.46 (9.31)	0.37	-0.46	39.78 (13.48)	-0.22	-0.38	36.64 (14.15)	0.59	-0.46
	Errors	0.33 (0.61)	1.25	0.88	0.6 (0.98)	1.04	-0.01	0.35 (0.49)	0.45	-0.79	0.64 (0.98)	0.57	-0.67
	Processed stimuli	35.46 (8.09)	-0.64	-0.95	36.06 (9.22)	0.28	-0.49	40.14 (13.36)	-0.19	-0.40	37.28 (14.00)	0.64	-0.42
Conners' Continuous Performance Test (III)	Response Style	54.93 (11.1)	-0.27	-0.87	60.66 (6.79)	0.49	-0.41	57.35 (13.39)	0.49	0.18	56.35 (13.35)	-0.17	-0.55
	Detectability	57.46 (7.69)	-0.71	-0.22	53.33 (9.57)	-0.18	-0.37	52.85 (8.06)	0.27	-0.47	53.28 (6.54)	0.61	0.44
	Omissions	59.66 (15.08)	0.52	-1.22	59.66 (16.31)	0.66	-0.45	55.07 (13.41)	1.09	0.35	55 (13.46)	0.94	0.27
	Perseverations	63.53 (17.77)	0.34	-1.59	57.46 (15.11)	1.00	0.03	56.5 (12.42)	1.14	0.52	57.71 (13.37)	0.95	0.03
	Mean Reaction Time	56.93 (12.37)	0.87	0.94	61.4 (11.45)	0.73	-0.25	61.71 (14.71)	0.38	-0.43	59.92 (17.34)	0.48	-0.51
	Standard Deviation of HRT	64.26 (15.31)	0.30	-1.34	65.2 (14.64)	0.11	-0.48	60.85 (13. 32)	0.33	-0.17	62.92 (13.29)	-0.11	-0.50
	Response Variability	60.66 (14.86)	0.59	-0.70	58.93 (10.47)	0.36	-0.60	58.38 (15.22)	0.67	-0.30	56.69 (12.12)	0.29	-0.53
	HRT Variability by ISI	61.13 (11.99)	0.39	-1.27	65.8 (15.27)	-0.56	-0.24	62 (17.43)	0.34	-0.56	62.14 (15.61)	0.01	-0.51
