# Supplementary material for: Electrophysiological Brain Changes Associated With Cognitive Improvement in a Pediatric Attention Deficit Hyperactivity Disorder Digital Artificial Intelligence-Driven Intervention: Randomized Controlled Trial
Source: J Med Internet Res. 2021 Nov 26;23(11):e25466. doi: 10.2196/25466 (PMC8665400; doi:10.2196/25466)

Figure S3. Efficacy outcome in Visuospatial Working Memory: mean change in Backward Span score from Corsi Block Tapping Test. Significant differences were found for Condition x Moment interaction effect in this cognitive measure (beta = -0.84, S.E = 0.38, t(27) = -2.24, p = 0.034)

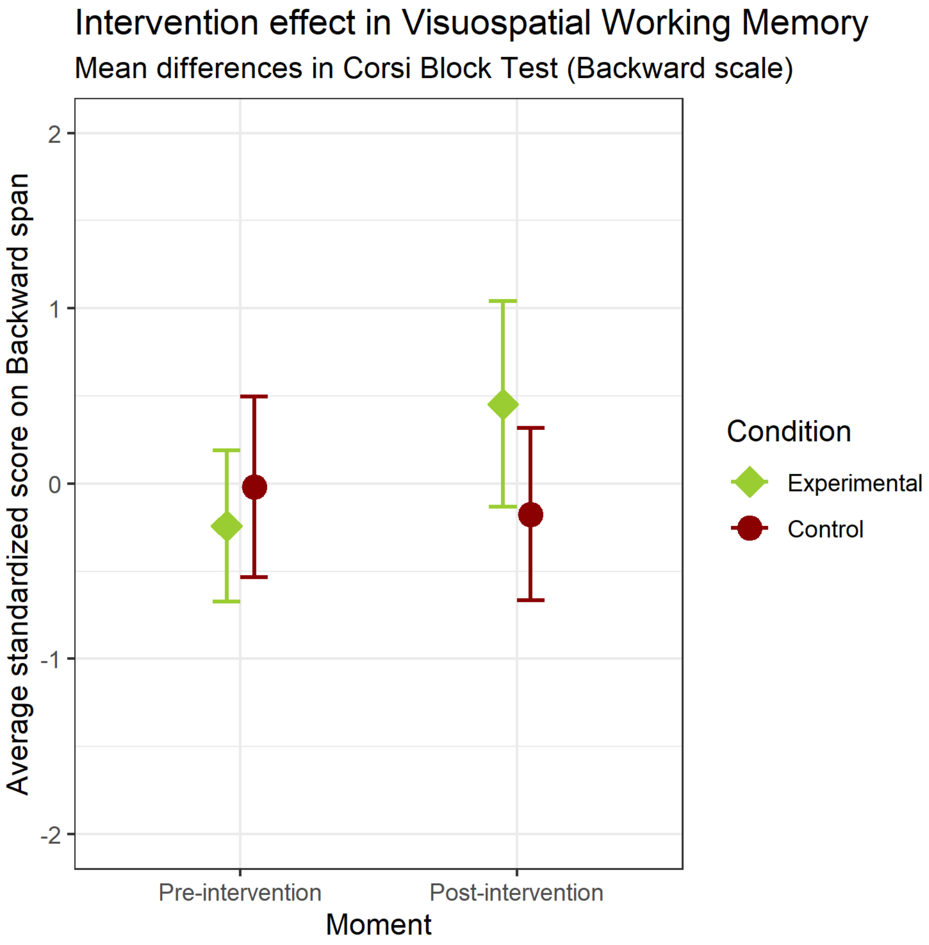

Supplement: Multimedia Appendix 13 [file jmir_v23i11e25466_app13.pdf]
