# Supplementary material for: Electrophysiological Brain Changes Associated With Cognitive Improvement in a Pediatric Attention Deficit Hyperactivity Disorder Digital Artificial Intelligence-Driven Intervention: Randomized Controlled Trial
Source: J Med Internet Res. 2021 Nov 26;23(11):e25466. doi: 10.2196/25466 (PMC8665400; doi:10.2196/25466)
Supplement: Multimedia Appendix 14 [file jmir_v23i11e25466_app14.pdf]

Table S6. Standardized mean differences for interaction effects in secondary outcome measures

Neuropsychological instrument	Outcome measure	Fixed effects	Standardized mean difference (95% CI)	t statistic	p value
Auditory Attention Test	Correct answers	(Intercept)	-0.16 (-0.69 - 0.37)	-0.59	0.558
		Treatment	0.29 (-0.47 - 1.05)	0.75	0.458
		Moment	0.01 (-0.55 - 0.58)	0.04	0.965
		Treatment x Moment	-0.01 (-0.81 - 0.79)	-0.03	0.975
	Commissions	(Intercept)	0.51 (0.01 - 1.02)	1.98	0.053
		Treatment	-0.40 (-1.12 - 0.31)	-1.10	0.276
		Moment	-0.79 (-1.49 - -0.09)	-2.19	0.037
		Treatment x Moment	0.36 (-0.65 - 1-36)	0.69	0.494
	Omissions	(Intercept)	0.30 (-0.23 - 0.83)	1.12	0.269
		Treatment	-0.37 (-1.12 - 0.38)	-0.97	0.338
		Moment	-0.23 (-0.81 - 0.35)	-0.77	0.446
		Treatment x Moment	0.05 (-0.77 - 0.88)	0.12	0.904
	Inhibition errors	(Intercept)	0.12 (-0.40 - 0.65)	0.46	0.644
		Treatment	0.18 (-0.56 - 0.92)	0.48	0.636
		Moment	-0.19 (-0.89 - 0.50)	-0.54	0.591
		Treatment x Moment	-0.47 (-1.46 - 0.52)	-0.93	0.360
Cognitive Flexibility Test	Correct answers	(Intercept)	-0.29 (-0.79 - 0.21)	-1.13	0.265
		Treatment	0.01 (-0.71 - 0.72)	0.01	0.989
		Moment	0.56 (0.11 - 1.02)	2.43	0.022
		Treatment x Moment	0.01 (-0.65 - 0.66)	0.03	0.978
	Commissions	(Intercept)	0.13 (-0.34 - 0.60)	0.55	0.585
		Treatment	0.50 (-0.17 - 1.18)	1.45	0.152
		Moment	-0.62 (-1,25 - 0.01)	-1.92	0.065
		Treatment x Moment	-0.27 (-1.18 - 0.64)	-0.57	0.572
	Omissions	(Intercept)	0.15 (-0.36 - 0.66)	0.57	0.574
		Treatment	-0.06 (-0.80 - 0.68)	-0.17	0.869
		Moment	-0.14 (-0.54 - 0.25)	-0.71	0.485
		Treatment x Moment	-0.19 (-0.77 - 0.38)	-0.67	0.510
	Inhibition errors	(Intercept)	0.18 (-0.30 - 0.66)	0.75	0.455
		Treatment	0.37 (-0.32 - 1.06)	1.06	0.296
		Moment	-0.57 (-1.25 - 0.10)	-1.66	0.109
		Treatment x Moment	-0.32 (-1.29 - 0.66)	-0.64	0.530
Digit Span Test	Forward correct answers	(Intercept)	-0.30 (-0.08 - 0.20)	-1.18	0.244
		Treatment	0.59 (-0.13 - 1.31)	1.60	0.118
		Moment	0.11 (-0.28 - 0.49)	0.54	0.593
		Treatment x Moment	-0.14 (0.70 - 0.41)	-0.51	0.614
	Forward span	(Intercept)	-0.40 (-0.88 - 0.08)	-1.64	0.109
		Treatment	0.80 (0.11 - 1.49)	2.26	0.029
		Moment	0.06 (-0.37 - 0.50)	0.29	0.772
		Treatment x Moment	-0.06 (-0.69 - 0.56)	-0.20	0.840
	Backward correct answers	(Intercept)	-0.11 (-0.63 - 0.40)	-0.44	0.664
		Treatment	-0.00 (-0.74 - 0.74)	-0.01	0.993
		Moment	0.09 (-0.42 - 0.60)	0.34	0.734
		Treatment x Moment	0.30 (-0.44 - 1.03)	0.78	0.440
	Backward span	(Intercept)	-0.24 (-0.75 - 0.28)	-0.90	0.373
		Treatment	0.28 (-0.46 - 1.02)	0.73	0.467
		Moment	0.32 (-0.29 - 0.93)	1.02	0.318
		Treatment x Moment	-0.23 (-1.11 - 0.65)	-0.52	0.609
Verbal fluency	Verbal Fluency Test (Semantic)	(Intercept)	0.01 (-0.51 - 0.53)	0.05	0.964
		Treatment	0.02 (-0.73 - 0.77)	0.05	0.962
		Moment	0.12 (-0.28 - 0.52)	0.58	0.565
		Treatment x Moment	-0.43 (-1.00 - 0.13)	-1.51	0.143
	Verbal Fluency Test (Phonological)	(Intercept)	-0.14 (-0.66 - 0.38)	-0.52	0.606
		Treatment	0.14 (-0.61 - 0.89)	0.36	0.720
		Moment	0.13 (-0.48 - 0.74)	0.42	0.678
		Treatment x Moment	-0.02 (-0.89 - 0.84)	-0.05	0.959
Inhibition Test	Errors	(Intercept)	-0.02 (-0.54 - 0.49)	-0.09	0.925
		Treatment	0.24 (-0.50 - 0.98)	0.64	0.528
		Moment	-0.20 (-0.60 - 0.20)	-0.97	0.341
		Treatment x Moment	0.04 (-0.55 - 0.62)	0.12	0.904
	Self-corrected errors	(Intercept)	0.01 (-0.51 - 0.52)	0.03	0.976
		Treatment	0.12 (-0.62 - 0.86)	0.33	0.745
		Moment	-0.26 (-0.76 - 0.18)	-1.16	0.255
		Treatment x Moment	0.26 (-0.37 - 0.89)	0.81	0.426
	Response time	(Intercept)	0.10 (-0.43 - 0.63)	0.37	0.716
		Treatment	0.21 (-0.55 - 0.96)	0.54	0.596
		Moment	0.22 (0.00 - 0.44)	1.91	0.088
		Treatment x Moment	-0.31 (-0.61 - -0.01)	-2.05	0.070
Corsi Block Tapping Test	Forward correct answers	(Intercept)	-0.36 (-0.86 - 0.15)	-1.38	0.176
		Treatment	0.56 (-0.17 - 1.29)	1.50	0.142
		Moment	0.42 (-0.04 - 0.88)	1.78	0.087
		Treatment x Moment	-0.51 (-1.18 - 0.16)	-1.50	0.146
	Forward span	(Intercept)	-0.31 (-0.82 - 0.20)	-1.19	0.240
		Treatment	0.48 (-0.25 - 1.21)	1.28	0.207
		Moment	0.50 (0.00 - 1.01)	1.97	0.059
		Treatment x Moment	-0.72 (-1.44 - 0.00)	-1.96	0.060
	Backward correct answers	(Intercept)	-0.20 (-0.70 - 0.30)	-0.80	0.430
		Treatment	-0.07 (-0.78 - 0.65)	-0.19	0.852
		Moment	0.63 (0.18 - 1.09)	2.73	0.011
		Treatment x Moment	-0.32 (-0.97 - 0.32)	-0.98	0.336
	Backward span	(Intercept)	-0.24 (-0.74 - 0.26)	-0.95	0.347
		Treatment	0.22 (-0.49 - 0.94)	0.61	0.545
		Moment	0.69 (0.17 - 1.21)	2.58	0.016
		Treatment x Moment	-0.85 (-1.59 - 0.10)	-2.23	0.034
	Correct answers	(Intercept)	-0.50 (-0.09 - -0.01)	-1.98	0.053
		Treatment	0.57 (-0.14 - 1.28)	1.56	0.125
		Moment	0.79 (0.24 - 1.35)	2.79	0.010
		Treatment x Moment	-0.71 (-1.50 - 0.08)	-1.76	0.091
	Repeated answers	(Intercept)	0.09 (-0.42 - 0.60)	0.35	0.728
		Treatment	0.16 (-0.57 - 0.89)	0.43	0.672

Card Classification Test	Repeated answers	Moment	-0.46 (-1.13 - 0.21)	-1.34	0.190
		Treatment x Moment	0.23 (-0.73 - 1.19)	0.47	0.640
	Unaccurate answers	(Intercept)	0.24 (-0.27 - 0.76)	0.92	0.361
		Treatment	-0.28 (-1.02 - 0.46)	-0.73	0.466
		Moment	-0.40 (-0.98 - 0.17)	-1.38	0.180
		Treatment x Moment	0.37 (-0.45 - 1.18)	0.88	0.388
	Total errors	(Intercept)	0.19 (-0.32 - 0.70)	0.75	0.458
		Treatment	-0.07 (-0.80 - 0.67)	-0.18	0.855
		Moment	-0.51 (-1.12 - 0.10)	-1.64	0.112
		Treatment x Moment	0.35 (-0.51 - 1.22)	0.80	0.431
Symbol Search Test	Correct answers	(Intercept)	-0.31 (-0.81 - 0.19)	-1.22	0.230
		Treatment	0.47 (-0.25 - 1.19)	1.27	0.214
		Moment	0.14 (-0.16 - 43)	0.91	0.371
		Treatment x Moment	0.08 (-0.35 - 0.50)	0.35	0.727
	Errors	(Intercept)	0.10 (-0.42 - 0.61)	0.36	0.719
		Treatment	-0.07 (-0.81 - 0.67)	-0.19	0.848
		Moment	-0.33 (-0.77 - 0.12)	-1.45	0.159
		Treatment x Moment	0.42 (-0.21 - 1.06)	1.31	0.200
	Processed stimuli	(Intercept)	-0.15 (-0.66 - 0.35)	-0.59	0.557
		Treatment	0.28 (-0.45 - 1.00)	0.75	0.459
		Moment	-0.14 (-0.58 - 0.29)	-0.65	0.521
		Treatment x Moment	0.37 (-0.25 - 1-00)	1.18	0.249
Digit Symbol Substitution Test	Correct answers	(Intercept)	-0.14 (-0.65 - 0.37)	-0.53	0.598
		Treatment	0.41 (-0.33 - 1.15)	1.09	0.282
		Moment	0.03 (-0.39 - 0.45)	0.14	0.892
		Treatment x Moment	-0.31 (-0.91 - 0.30)	-1.00	0.328
	Errors	(Intercept)	-0.19 (-0.70 - 0.32)	-0.74	0.465
		Treatment	0.03 (-0.71 - 0.77)	0.08	0.935
		Moment	0.34 (-0.27 - 0.95)	1.10	0.281
		Treatment x Moment	0.02 (-0.85 - 0.90)	0.05	0.957
	Processed stimuli	(Intercept)	-0.15 (-0.67 - 0.36)	-0.15	0.561
		Treatment	0.42 (-0.32 - 1.16)	0.42	0.275
		Moment	0.05 (-0.37 - 0.48)	0.05	0.808
		Treatment x Moment	-0.31 (-0.92 - 0.31)	-0.31	0.334
Conners' Continuous Performance Test (III)	Response Style	(Intercept)	-0.21 (-0.72 - 0.30)	-0.82	0.418
		Treatment	0.21 (-0.52 - 0.95)	0.57	0.571
		Moment	0.51 (0.07 - 0.95)	2.27	0.032
		Treatment x Moment	-0.60 (-1.23 - 0.04)	-1.85	0.075
	Detectability	(Intercept)	0.39 (-0.11 - 0.90)	44.56	0.133
		Treatment	-0.57 (-1.30 - 0.16)	44.56	0.131
		Moment	-0.51 (-1.04 - 0.01)	27.00	0.067
		Treatment x Moment	0.56 (-0.19 - 1.32)	27.00	0.155
	Omissions	(Intercept)	0.15 (-0.36 - 0.67)	0.58	0.563
		Treatment	-0.31 (-1.05 - 0.43)	-0.83	0.410
		Moment	0.00 (-0.50 - 0.50)	0.00	1.000
		Treatment x Moment	0.00 (-0.73 - 0.72)	-0.01	0.990
	Perseverations	(Intercept)	0.31 (-0.19 - 0.83)	1.22	0.230
		Treatment	-0.48 (-1.21 - 0.26)	-1.27	0.209
		Moment	-0.41 (-1.00 - 0.18)	-1.36	0.185
		Treatment x Moment	0.49 (-0.36 - 1.35)	1.13	0.267
	Mean Reaction Time	(Intercept)	-0.21 (-0.73 - 0.30)	-0.83	0.409
		Treatment	0.34 (-0.40 - 1.09)	0.91	0.366
		Moment	0.32 (-0.16 - 0.81)	1.31	0.202
		Treatment x Moment	-0.45 (-1.15 - 0.24)	-1.27	0.214
	Standard Deviation of HRT	(Intercept)	0.06 (-0.45 - 0.58)	0.25	0.806
		Treatment	-0.24 (-0.99 - 0.50)	-0.65	0.521
		Moment	0.07 (-0.54 - 0.68)	0.22	0.831
		Treatment x Moment	0.08 (-0.80 - 0.96)	0.18	0.856
	Response Variability	(Intercept)	0.14 (-0.37 - 0.66)	0.56	0.580
		Treatment	-0.16 (-0.92 - 0.59)	-0.42	0.677
		Moment	-0.13 (-0.73 - 0.48)	-0.42	0.677
		Treatment x Moment	0.04 (-0.87 - 0.95)	0.09	0.926
	HRT Variability by ISI	(Intercept)	-0.11 (-0.63 - 0.40)	-0.42	0.673
		Treatment	0.06 (-0.68 - 0.80)	0.15	0.878
		Moment	0.31 (-0.26 - 0.89)	1.08	0.291
		Treatment x Moment	-0.30 (-1.13 - 0.52)	-0.73	0.474
