# Supplementary material for: Electrophysiological Brain Changes Associated With Cognitive Improvement in a Pediatric Attention Deficit Hyperactivity Disorder Digital Artificial Intelligence-Driven Intervention: Randomized Controlled Trial
Source: J Med Internet Res. 2021 Nov 26;23(11):e25466. doi: 10.2196/25466 (PMC8665400; doi:10.2196/25466)
Supplement: Multimedia Appendix 15 [file jmir_v23i11e25466_app15.pdf]

Table S3. Pre-post standardized mean differences (Hedges' g) per Treatment group

Main outcome		Experimental			Control		
		Pre Mean (SD)	Post Mean (SD)	Hedges g*	Pre Mean (SD)	Post Mean (SD)	Hedges g*
Commisions score from CPT-III		53.86 (8.37)	47.8 (8.21)	-0.62	48.78 (7.52)	49.64 (7.32)	0.10
Secondary outcomes							
EDAH scales	Hyperactivity score	12.46 (3.39)	8.66 (3.47)	-0.93	11.78 (3.76)	8.5 (3.54)	-0.76
	Inattention score	13.33 (3.19)	9.26 (2.78)	-1.14	11.85 (4.07)	9.14 (3.57)	-0.60
	score	25 (5.49)	17.93 (4.78)	-1.16	23.64 (6.33)	17.64 (6.54)	-0.78
	Behavioural Disorders score	19.46 (7.94)	10.06 (6.11)	-1.12	17.92 (7.51)	10.78 (6.07)	-0.88
BRIEF	Inhibition score	20.8 (3.85)	20 (5.6)	-0.14	22 (4.81)	20.35 (4.81)	-0.29
	Flexibility score	16.8 (2.70)	15.6 (3.5)	-0.32	15.71 (3.53)	16.14 (3.10)	0.11
	Emotional Control score	21.66 (4.54)	20.06 (6.20)	-0.25	21.85 (4.24)	20.57 (3.56)	-0.28
	Initiate score	17.66 (2.28)	17.06 (3.08)	-0.19	17.07 (3.17)	17.5 (4.25)	0.10
	Working Memory score	25.73 (2.84)	24.46 (3.66)	-0.33	24.14 (4.45)	23.42 (4.55)	-0.13
	Planification score	29.8 (3.66)	28.53 (4.34)	-0.27	27.78 (4.11)	27.57 (4.71)	-0.04
	Organization score	13.4 (3.97)	12.73 (3.08)	-0.14	14.28 (2.84)	14 (3.94)	-0.07
	Monitoring score	21.06 (2.28)	21.06 (5.10)	0.00	20.5 (1.50)	20 (3.01)	-0.18
Auditory Attention Test	Correct answers	27.28 (3.81)	27.13 (2.79)	-0.04	28 (3.23)	28 (2.85)	0.00
	Commissions	4.28 (2.64)	2.46 (1.50)	-0.71	3.35 (2.67)	2.35 (1.90)	-0.36
	Omissions	2.85 (3.39)	2.4 (2.82)	-0.12	2 (2.25)	1.5 (2.76)	-0.17
	Inhibition errors	2.07 (1.73)	1.66 (1.71)	-0.20	2.42 (2.95)	1.07 (1.43)	-0.49
Cognitive Flexibility Test	Correct answers	27.33 (4.25)	29.93(4.69)	0.49	27.35 (4.68)	30 (4.47)	0.49
	Commissions	4.2 (2.93)	1.8 (2.04)	-0.80	6.14 (5.58)	2.71 (2.94)	-0.65
	Omissions	5.93 (4.21)	5.26 (4.62)	-0.13	5.64 (5.19)	4.07 (4.82)	-0.26
	Inhibition errors	3.53 (3.02)	1.6 (1.68)	-0.67	4.78 (4.96)	1.78 (2.19)	-0.66
Digit Span Test	Forward correct answers	6.46 (0.91)	6.66 (1.23)	0.16	7.57 (2.44)	7.5 (2.40)	-0.02
	Forward span	4.46 (0.51)	4.53 (0.74)	0.09	5.28 (1.13)	5.28 (1.32)	0.00
	Backward correct answers	5.93 (1.33)	6.06 (0.96)	0.10	5.92 (1.59)	6.5 (1.99)	0.27
	Backward span	3.26 (0.70)	3.53 (0.63)	0.33	3.5 (1.01)	3.57 (1.01)	0.06
(Semantic)	Correct answers	27.93 (8.68)	29.64 (5.37)	0.20	28.07 (6.87)	25.71 (8.60)	-0.25
(Phonological)	Correct answers	14.2 (6.13)	15.14 (5.46)	0.14	14.92 (4.63)	15.5 (5.28)	0.10
Inhibition Test	Errors	3.06 (4.63)	1.93 (1.98)	-0.27	4.42 (8.14)	3.5 (6.59)	-0.11
	Self-corrected errors	4.06 (4.47)	3 (2.85)	-0.24	4.57 (4.46)	4.57 (4.58)	0.00
	Response time	24.6 (8.23)	89.08 (48.06)	1.56	56.66 (46.77)	84 (40.63)	0.53
Corsi Block Tapping Test	Forward correct answers	6.6 (1.91)	7.6 (2.13)	0.42	7.92 (2.75)	7.71 (2.67)	-0.07
	Forward span	4.86 (1.06)	5.53 (1.45)	0.44	5.5 (1.40)	5.21 (1.36)	-0.17
	Backward correct answers	5.8 (2.14)	7.28 (2.43)	0.55	5.64 (1.94)	6.35 (2.46)	0.27
	Backward span	4.26 (1.16)	5.21 (1.57)	0.58	4.57 (1.34)	4.35 (1.27)	-0.14
Card Classification Test	Correct answers	3.2 (2.30)	5.28 (2.58)	0.72	4.64 (2.61)	4.85 (2.45)	0.07
	Repeated answers	2.13 (1.50)	1.28 (0.99)	-0.56	2.42 (1.65)	2 (2.82)	-0.16
	Unaccurate answers	1.8 (2.51)	1.07 (1.43)	-0.30	1.28 (1.71)	1.21 (1.62)	-0.04
	Total errors	3.93 (3.53)	2.35 (1.82)	-0.47	3.71(2.89)	3.21 (4.07)	-0.12
Symbol Search Test	Correct answers	22.66 (5.32)	23.4 (5.15)	0.12	25.14 (4.89)	26.28 (5.67)	0.18
	Errors	3.13 (5.13)	1.26 (2.7)	-0.38	2.71 (5.09)	3.28 (8.87)	0.07
	Processed stimuli	25.8 (6.65)	24.73 (4.38)	-0.16	27.85 (7.12)	29.57 (10.40)	0.16
Digit Symbol Substitution Test	Correct answers	35.13 (8.04)	35.46 (9.31)	0.03	39.78 (13.48)	36.64 (14.15)	-0.19
	Errors	0.33 (0.61)	0.6 (0.98)	0.27	0.35 (0.49)	0.64 (0.98)	0.32
	Processed stimuli	35.46 (8.09)	36.06 (9.22)	0.06	40.14 (13.36)	37.28 (14.00)	-0.18
Conners' Continuous Performance Test (III)	Response Style	54.93 (11.1)	60.66 (6.79)	0.53	57.35 (13.39)	56.35 (13.35)	-0.06
	Detectability	57.46 (7.69)	53.33 (9.57)	-0.40	52.85 (8.06)	53.28 (6.54)	0.05
	Omissions	59.66 (15.08)	59.66 (16.31)	0.00	55.07 (13.41)	55 (13.46)	0.00
	Perseverations	63.53 (17.77)	57.46 (15.11)	-0.31	56.5 (12.42)	57.71 (13.37)	0.08
	Mean Reaction Time	56.93 (12.37)	61.4 (11.45)	0.32	61.71 (14.71)	59.92 (17.34)	-0.09
	Standard Deviation of HRT	64.26 (15.31)	65.2 (14.64)	0.05	60.85 (13. 32)	62.92 (13.29)	0.13
	Response Variability	60.66 (14.86)	58.93 (10.47)	-0.11	58.38 (15.22)	56.69 (12.12)	-0.10
	HRT Variability by ISI	61.13 (11.99)	65.8 (15.27)	0.29	62 (17.43)	62.14 (15.61)	0.01

*Negative Hedges g indicates that post-treatment score is lower than pre-treatment.
