# Supplementary material for: Electrophysiological Brain Changes Associated With Cognitive Improvement in a Pediatric Attention Deficit Hyperactivity Disorder Digital Artificial Intelligence-Driven Intervention: Randomized Controlled Trial
Source: J Med Internet Res. 2021 Nov 26;23(11):e25466. doi: 10.2196/25466 (PMC8665400; doi:10.2196/25466)
Supplement: Multimedia Appendix 16 [file jmir_v23i11e25466_app16.pdf]

Table S7. Descriptives, t statistics, p-values, confidence intervals and respondent percentage per score in EDAH scale.

| Clinical outcome measure | Intervention | N | Mean pre (SD) | Mean post (SD) | Test Statistic (T) | p | 95% CI [U,L] | Effect size | Number of patient cut point changing | % cut point changing by group |
|---|---|---|---|---|---|---|---|---|---|---|
| Hiperactivity | Experimental | 15 | 93.6 (9.71) | 81 (21.15) | 2.12 | 0,05* | [ 0.00, 1.12] | 0.57 | 4 | 27% |
| | Control | 14 | 91.28 (11.33) | 80 (20.18) | 2.03 | 0.06 | [-0.03, 1.14] | 0.56 | 4 | 29% |
| Attention Deficit | Experimental | 15 | 97.86 (2.16) | 89.13 (8.38) | 4.56 | < 0,001** | [0.06, 1.21] | 0.65 | 7 | 47% |
| | Control | 14 | 92.57 (13.35) | 83.35 (16.92) | 2.9 | 0,01** | [0.18, 1.43] | 0.81 | 4 | 22% |
| Behavior disorder | Experimental | 15 | 94.66 (12.45) | 80.5 (23.51) | 3.78 | < 0,001** | [0.36, 1.64] | 1.01 | 8 | 53% |
| | Control | 14 | 89.5 (18.49) | 79.78 (26.86) | 1.46 | 0.16 | [-0.17, 0.97] | 0.41 | 1 | 7% |
| Global | Experimental | 15 | 97.86 (3.60) | 88 (9.57) | 4.00 | < 0,001** | [0.40, 1.71] | 1.07 | 9 | **60%** |
| | Control | 14 | 95.71 (6.85) | 83.71 (20.87) | 2.55 | 0,02* | [0.09, 1.30] | 0.71 | 3 | **21%** |

* $p < 0.05$; ** $p < 0.0125$ under Bonferroni correction (0.05/4)
